# Geography is not destiny: A quantitative test of Diamond's axis of orientation hypothesis

**DOI:** 10.1017/ehs.2023.34

**Published:** 2024-01-04

**Authors:** Angela M. Chira, Russell D. Gray, Carlos A. Botero

**Affiliations:** 1 Department of Biology, Washington University in St Louis, St Louis, Missouri, USA; 2 Department of Linguistic and Cultural Evolution, Max Planck Institute for Evolutionary Anthropology, Leipzig, Germany; 3 School of Psychology, University of Auckland, Auckland 1142, New Zealand; 4 Department of Integrative Biology, The University of Texas at Austin, Austin, Texas, USA

**Keywords:** Axis of orientation hypothesis, cultural evolution, cultural biogeography

## Abstract

Jared Diamond suggested that the unique East–West orientation of Eurasia facilitated the spread of cultural innovations and gave it substantial political, technological and military advantages over other continental regions. This controversial hypothesis assumes that innovations can spread more easily across similar habitats, and that environments tend to be more homogeneous at similar latitudes. The resulting prediction is that Eurasia is home to environmentally homogenous corridors that enable fast cultural transmission. Despite indirect evidence supporting Diamond's influential hypothesis, quantitative tests of its underlying assumptions are currently lacking. Here we address this critical gap by leveraging ecological, cultural and linguistic datasets at a global scale. Our analyses show that although societies that share similar ecologies are more likely to share cultural traits, the Eurasian continent is not significantly more ecologically homogeneous than other continental regions. Our findings highlight the perils of single factor explanations and remind us that even the most compelling ideas must be thoroughly tested to gain a solid understanding of the complex history of our species.

## Graphical abstract

**Figure:**
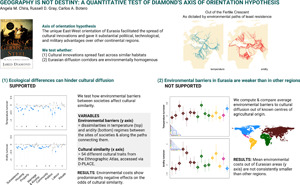

**Social media summary:** Environmental barriers may limit cultural spread but do not consistently favour Eurasia.

## Introduction

In *Guns, germs, and steel*, Jared Diamond speculated that ecological biases in the spread of cultural innovations contributed to the different rates at which societies developed in different regions of our world (Diamond, 1997, 2002). Specifically, he noted that while the predominant axis of orientation of Africa and the Americas is North–South, that of Eurasia is mainly East–West, suggesting greater homogeneity in day length, climate and/or available habitats within the Eurasian continent. Based on this observation, Diamond posited that geography could have facilitated the transmission and successful implementation of cultural innovations in Eurasia, particularly those related to the domestication of flora and fauna. Some telling examples support these ideas. For example, although agriculture spread rapidly within Southern Asia, Europe and the Indus Valley, it spread relatively slowly out of Mexican, Eastern North American, South American and African points of origin (Diamond, 1997; Stephens et al., 2019; Zohary et al., 2012). Other fundamental innovations followed a similar trend. Wheels and alphabetic writing, respectively, spread rapidly from South–West Asia to Eurasia and from the Fertile Crescent to Carthage and the Indian subcontinent but failed to spread from Mesoamerica to South America (Diamond, 1997). Importantly, the rapid spread of crucial innovations related to domestication in Eurasia was followed by the development of large, dense, sedentary, and stratified societies. Thus, in Diamond's own words, geography may have ‘turned the fortunes of history’ and shaped inter-continental differences in technological, political and military dominance (Diamond, 1997).

Diamond's biogeographic insights and remarkable scope have received much attention. His book, *Guns, germs, and steel* (Diamond, 1997) won the 1998 Pulitzer Prize in General Nonfiction (https://www.pulitzer.org/winners/jared-diamond) and it was the base of a three-part National Geographic Special aired in 2005. However, his thesis has also been heavily criticised for being Eurocentric and for failing to recognise the importance of historical contingency and cultural factors (Blaut, 1999; York & Mancus, 2007). Further, in relation to the axis of orientation hypothesis, as this influential idea is now known, his critics have noted the inherent risks of downplaying exceptions when extrapolating from hand-picked examples of fast cultural transmission within similar environments (Blaut, 1999). The assumption that environmental conditions are significantly more homogeneous in Eurasia than in other regions of the world is also problematic. For example, the Himalayan plateau, the Arabian desert and the Mediterranean are physically connected areas within the Eurasian continental landmass that occur at similar latitudes but exhibit pronounced ecotones and elevational gradients in abiotic conditions. Another potential issue with Diamond's proposition is that although domesticated species undoubtedly show ecological limits, their potential for adaptation can be wide and can often be extended with the assistance of human modifications of the environment (Blaut, 1999; Fuller & Qin, 2009). Despite these critiques, Diamond's ideas have received some theoretical and empirical support. For example, Olsson and Hibbs (2005) build on *Guns, germs, and steel* to investigate the role of biogeographic conditions on the transition to sedentary agriculture and argue for a long-lasting influence of early ecological biases (such as size of continent and major continental axis, climate type and the number of plant and animal species suited for domestication). Further, historical and modern states are predominantly oriented East–West (Currie & Mace, 2009; Turchin et al., 2006), and linguistic diversity tends to be more pronounced across longitudinal than latitudinal gradients – i.e. culture probably diffuses more easily on an East–West than on a North–South axis (Güldemann & Hammarström, 2020; Laitin et al., 2012). Quantitative evidence supporting Diamond's idea and particularly its core assumptions is nevertheless scarce. Crucially, it remains to be established whether environmental barriers associated with latitude significantly hinder the spread of cultural traits and whether continental-scale differences in the strength of such environmental barriers actually follow Diamond's expectation, i.e. whether ecological facilitation of cultural spread is indeed higher in the Eurasian landmass.

**Figure 1.**
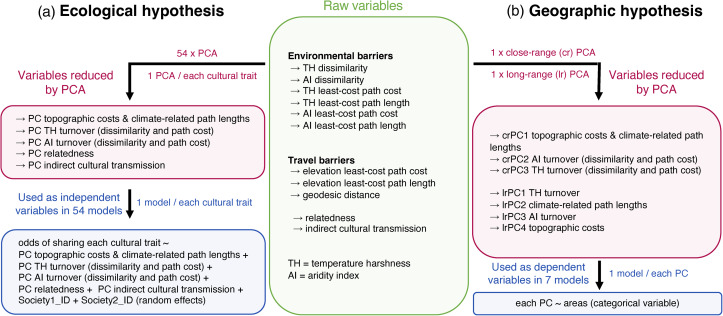
General workflow for testing the (a) ecological and (b) geographic premises of Diamond's hypothesis. A global principal component analysis (PCA) at 0.5 × 0.5° resolution is first used to derive variation in temperature harshness (TH) and aridity index (AI) around the world. These climatic variables are used to compute environmental barriers to cultural spread between pairs of societies. (a) Environmental and travel barriers, along with metrics of relatedness and cultural transmission from outside the pair (green box) are run through PCAs (one for each cultural trait) to extract five principal components (PCs, burgundy box). These PCs are then used as independent variables in 54 separate models predicting the odds of sharing key cultural practices between societies in pairs (one model for each cultural trait; blue box). (b) Environmental and travel costs associated with cultural spread out of the location of each society in an agricultural centre of origin are run through two separate PCAs to cover close- and long- range spatial scales (green to burgundy boxes). We then extract three close-range (cr) and four long-range (lr) PCs as estimates of environmental barriers and use them as response variables in seven separate models (i.e. a model for each PC; blue box). In all models, the independent factor is a categorical variable showing the membership of societies to various areas of agricultural origin.

Operationalising Diamond's hypothesis has proved difficult in part because it requires the integration of vast amounts of data from multiple sources that were not easily accessible in the past. Here we explicitly test the most critical ecological and geographic assumptions of Diamond's hypothesis through quantitative analyses that leverage a comprehensive set of now publicly available data on global differences in culture, languages, and ecology (Gray, 1999; Kirby et al., 2016; Murdock, 1967). We first use cultural and ecological data to identify the potential for environmental parameters to influence the ease of cultural transmission (Figure 1a, Figure S1a). While Diamond primarily frames his arguments around cultural diffusion (i.e. the acquisition of innovations and knowledge from neighbouring societies, Edmonson, 1961), the spread of innovations with the movement of peoples (demic diffusion, Ammerman & Cavalli-Sforza, 1971) was a fundamental part of the Neolithic transition (i.e. a period of time that is core to the axis of orientation hypothesis; Diamond & Bellwood, 2003; Fort, 2012). We quantify patterns of cultural spread by using cultural similarity between pairs of societies. By using this metric, our analyses essentially capture patterns that can result from both demic and cultural diffusion. Further, we focus on geographic and environmental barriers that include barriers related directly to human movement (such as travel costs), as well as environmental barriers that would hinder both cultural diffusion (spread and successful implementation of cultural innovations) and the migration and settlement of peoples. Specifically, we quantify pairwise dissimilarity in temperature, aridity, and topography between societies by measuring not only how different their sites of residence are, but also, how environmental parameters change along the paths that connect them. We then test whether the odds of sharing key cultural traits between societies are negatively affected by the magnitude of environmental dissimilarities, as proposed in Diamond's thesis. Finally, we quantify and contrast ecological barriers within corridors of cultural transmission in different continents. Given that the spread of agricultural innovations is such a pervasive example in Diamond's book, we compute and compare ecological barriers out of known centres of agricultural origin (Figure 1b and Figure S1b). Based on Diamond's arguments, we expect to see consistently smaller ecological barriers in the potential pathways of agriculture transmission within Eurasia than in other regions of our world.

## Methods

### Ecological hypothesis: ecology can influence the potential for cultural transmission

#### Quantifying cultural sharing

We used the D-PLACE database (Gray, 1999; Kirby et al., 2016; Murdock, 1967) to collect data on the cultural makeup of 1094 mainland traditional societies (Figure S2), defined as groups of people at a focal location whose language and cultural traditions set them apart from neighbouring groups (Kirby et al., 2016). Out of the total 1291 societies represented in the Ethnographic Atlas dataset within D-PLACE, we excluded societies located on islands, based on the geographic positions of societies in Glottolog (Hammarström et al., 2019) and using the island polygons described in Weigelt et al. (2013) as reference for continental and island terrain. We considered 67 cultural traits related to subsistence, house construction, property rules, community organisation, marriage and kinship, politics, class, labour, and ritual practices (Data S1). For each cultural trait, we scored whether a pair of societies, our unit of analysis, exhibited the same or different trait values. For example, each society exhibits a dominant mode of subsistence (dependence predominantly on gathering, hunting, fishing, pastoralism or agriculture), and two societies are identified as sharing the dominant subsistence mode if they both rely predominantly on, for example, the gathering of wild plants and small land fauna relative to other subsistence modes. In practice, this means that a pair of societies shares a cultural trait if societies share the codes allocated for that trait in the D-PLACE database. For some traits, we modified the original D-PLACE coding to better reflect the question of interest. For example, we considered societies that rely on casual, extensive, intensive agriculture (or any combination of these) as sharing an agricultural mode of subsistence. Further, we did not consider societies in which a cultural trait of interest is absent, e.g. when looking at whether societies share the pattern for the largest matrilinear group, we excluded societies in which matrilinear groups are absent. We also did not consider societies with missing cultural data. Lastly, we excluded cultural traits with only two discrete code categories. Data S1 contains a list with all traits for which we scored cultural similarity (a total of 67) alongside their code values, number of categories, and a notes tab justifying the codes used. All the traits we used are discrete.

#### Quantifying environmental barriers to cultural transmission

We built pairs of societies by considering each society and its closest 100 neighbours based on geodesic distances. To determine geodesic distances, we rely on the coordinates of societies as they are listed in Glottolog (Hammarström et al., 2019). From the initial 100 pairs for each society, we select the pairs for which we have cultural data. We then evaluated whether the odds of sharing the same values for a given cultural trait are impacted by environmental parameters associated with latitude. Following Diamond's hypothesis, we focus on dissimilarities in environmental conditions at the locations of societies in pairs, as well as on the magnitude of ecological barriers that the use of a cultural trait may have encountered as it expanded its range. If Diamond's ecological premise is supported by the data, we expect that the odds of cultural similarity will be negatively impacted by high environmental dissimilarities between the home ranges of the societies in the pair, and by high environmental heterogeneity along the paths that connect them. We follow Diamond's original framework and primarily focus on ecological drivers of cultural transmission; we do, however, extend our analyses to include topographic factors, i.e. barriers associated with the movement of humans.

Environmental costs to cultural transmission between pairs of societies were estimated from data on 100+ year time series on local mean, variability and predictability of temperature and precipitation, as available in the EcoClimate database (Lima-Ribeiro et al., 2015). These climatic variables exhibit pronounced latitudinal gradients and are heavily featured in Diamond's (1997) book. Given that the patterns of geographic variation in these parameters are highly correlated, we reduced them to two composite variables through a global principal component analysis (PCA) at a 0.5 × 0.5° resolution (Botero et al., 2014a; Table S1). Only land cells were included. The resulting components captured global gradients in the variation of ‘temperature harshness’ (PC1) and ‘aridity index’ (PC2). PC1 values reflect a continuum from cold, unpredictable and variable temperatures to warm, predictable and invariable conditions. PC2 values reflect a continuum from arid, unpredictable and variable precipitation to humid, predictable and invariable regimes (Figure S3).

**Figure 2.**
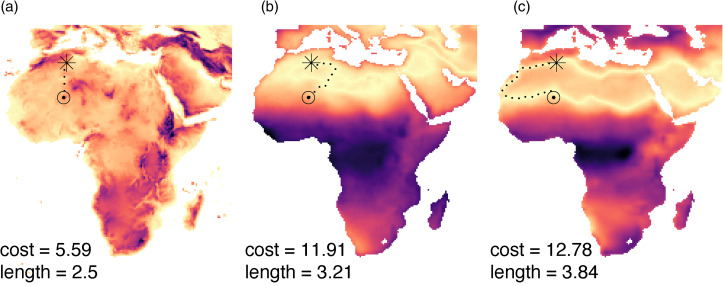
Example of (a) topographic, (b) temperature, and (c) aridity least-cost paths between the locations of two societies (starting point is marked by a star). Dark colours on the cost maps indicate cells of high cost – i.e. high elevation (a) or high differences in temperature harshness (b) and aridity index (c) as compared with the starting point. Environmental heterogeneity along the least-cost path connecting two societies in a pair is estimated as the log value of accumulated cost and log value of path length.

Environmental dissimilarity between societies in pairs was estimated as the absolute difference in temperature harshness and aridity index, respectively, between the geographic positions of societies (Figure S4). We estimated environmental heterogeneity in the intervening space between societies in pairs using least-cost path algorithms. Specifically, starting from each society in a pair, we computed the path toward the other society that minimised aridity index differentials with the starting location (Alberti, 2019; van Etten, 2017; Figure 2, see Figure S4 for details about the least-cost path algorithm). A high accumulated cost along this path indicates high dissimilarity in aridity regime from the starting location to the destination society. Similarly, longer paths indicate that longer, hence costlier, diffusion (and travel) routes are needed to achieve a minimal aridity regime difference between the starting and destination societies. For each society pair ‘AB’, we computed the paths of least resistance starting from the coordinates of A towards the coordinates of B and vice-versa. We used the mean accumulated cost values on the paths A to B and B to A, and the mean length of paths A to B and B to A as estimates of environmental heterogeneity in the intervening space between A and B (Figure S4). We then repeated this algorithm to estimate the path of minimal temperature harshness differences. Lastly, we measured the length and cost of least-cost topographic paths (as measured through changes in slope of the terrain), alongside geodesic distances as indicators of barriers to cultural similarity related to human travel costs (see Figure S4 for details). All analyses included geographic correction to account for the spatial distortion of latitude/longitude map projections in the WGS 84 coordinate system (van Etten, 2017).

#### Common ancestry and potential of indirect transmission from neighbouring cultural groups

Patterns of cultural similarity between societies are known to be shaped by common ancestry and horizontal diffusion (Mace & Holden, 2005; Mace & Jordan, 2011). We thus accounted for the possibility that societies in a pair share the same cultural trait values owing to shared ancestry by considering relatedness as a predictor in our models. Language phylogenies are commonly used to account for cultural similarity owing to shared ancestry (Evans et al., 2021). This is because languages are a good representation of the history of expansions and splits of cultural groups and thus reasonably estimate population history. We estimated relatedness using a recent Bayesian phylogeny of all extant languages (Bouckaert et al., 2022, preprint). This super-tree incorporates information on established language classification within families, language location, historical, linguistic, and archaeological evidence for the deeper splits, as well as genetic and archaeological evidence about the colonisation of the world by humans. The Bayesian approach incorporates uncertainty about the relationships among languages in the form of a posterior distribution of possible trees. For the analyses in the main text, we used TreeAnnotator to build a maximum clade credibility tree, setting node heights to common ancestor (Drummond et al., 2012). We prune this tree for the societies in our dataset (Figure S5), and then compute our relatedness metric as the phylogenetic cophenetic distance (based on branch lengths) to the nearest common ancestor of the two societies in a pair9) (we used the R package {ape}, Paradis & Schilep, 2019). To test the robustness of our results to phylogenetic uncertainty, we also run the analyses using a randomly drawn sample of 50 trees from the posterior of alternative trees (Data S3).

Our analyses are focused on cultural similarity between pairs of societies. When two societies share the same cultural trait value, they could be doing so because of cultural transmission between them. Alternatively, cultural transmission could have happened between each of the two societies in a pair and other close-by neighbours. We note here that Diamond's thesis is not reliant on the assumption of direct transmission between pairs of societies. However, we believe that comparing society pairs facilitates a principled definition of cultural influences and enables a more straightforward interpretation of results, particularly when compared with difficult to define cultural ‘neighbours’. Thus, our models ask: do geographical and environmental barriers promote cultural similarity between pairs of societies above and beyond what is expected from shared ancestry or transmission from other close-by societies? In practice, for each society, we calculated the proportion of the five closest neighbours (based on geodesic distances) for which cultural data exist in the D-PLACE database that share the trait of its pair member (following Botero et al., 2014b). If a high percentage of nearby neighbours of society ‘A’ express the trait of society ‘B’ (pair member of ‘A’), there is a high chance that ‘A’ has the same trait as ‘B’ not because of cultural transmission between ‘B’ and ‘A’, but because of cultural transmission from the close neighbours of ‘A’ to ‘A’. That is, ‘A’ got the trait value of ‘B’ because of transmission within its close neighbourhood (Figure S6). For each pair ‘AB’, we compute the potential for indirect transmission from other cultural groups for society ‘A’, then for ‘B’, and then average the two values. We assume that higher values of this predictor indicate that a society could have picked a shared cultural trait from its immediate neighbours rather than from the other society in the pair (Botero et al., 2014b). This is a conservative assumption, as it equates a pattern of spatial clustering with the mechanism of horizontal diffusion, even though in reality we cannot know whether cultural transmission actually happened or not (Evans et al., 2021). As a sensitivity analysis, we also computed our metric of cultural transmission from other groups using each society's 10 closest neighbours, regardless of whether these neighbouring societies had data in the D-PLACE dataset (i.e. what proportion of the closest 10 neighbours of ‘A’ have the trait value of ‘B’?, and vice-versa). In this way, the selection of which societies to consider as the ‘close neighbourhood’ was not affected by sampling in the D-PLACE dataset. Pairs in which neighbourhood structure could not be estimated were discarded (if cultural data were available for at least three out of the 10 nearest neighbours for both societies in the pair, the pair was kept in the analyses).

#### Modelling cultural sharing as a function of environmental dissimilarities

We used logistic regressions to correlate the odds of societies sharing cultural traits with our initial 11 candidate drivers of cultural sharing: temperature and aridity dissimilarity, the length and accumulated cost along the least-cost path for human travel, the geodesic distance, the length and accumulated cost along the temperature and aridity least-cost paths, shared ancestry, and cultural transmission from other nearby societies (Figure 1a, Figure S1a, the green ‘Raw variables’ box). To recapitulate, in our models, cultural sharing is a binary variable, with ‘1’ indicating the two societies are culturally similar (i.e. the pair shares the codes allocated for that trait in the D-PLACE database), and ‘0’ indicating the reverse. We set the error family as Bernoulli. However, most such models suffered from strong multicollinearity (variance inflation factors > 5; Zuur et al., 2010). Thus, we reduced the initial set of predictors to five composite variables via a varimax-rotated PCA (Figure 1a and Figure S1a, the burgundy ‘Variables reduced by PCA’ box). The PCAs were run separately for each cultural trait because the potential for transmission from nearby neighbours is based on patterns that are specific to each trait (Table S2 shows how each raw variable connects to a component in each of these PCAs). For each trait, we used five composite variables as predictors for cultural sharing in mixed-effects models that included the identities of societies in pairs as random effects (modelled as random intercepts; Figure 1a and Figure S1a, blue box). Thus, we asked whether the odds of cultural sharing in pairs (i.e. whether or not societies in a pair share the same focal cultural trait value) were predicted by five candidate drivers: (i) temperature turnover (component capturing variation in temperature harshness heterogeneity at the location of societies in pairs and along the temperature-based least-cost paths); (ii) aridity turnover (capturing aridity index heterogeneity at the location of societies in pairs and along the aridity-based least-cost paths); (iii) costs to human movement (capturing topographic costs and climate-related path lengths); (iv) common ancestry; and (v) transmission from other close-by neighbours (Figure 1a and Figure S1a, blue box). In our models, higher principal component values show higher environmental and travel barriers. Thus, if Diamond's ecological assumption is supported, we expect to see negative associations (negative estimated effects) between cultural similarity and temperature turnover, aridity turnover, and travel costs. We used the function glmer() to run the models in R (Bates et al., 2015). Spatially neighbouring data points could show similarity in predictors, response variables, and/or the relationship between the two (i.e. the assumption of independent residuals is breached). Models that suffer from spatial autocorrelation can show inflated Type I errors. We thus used Moran's I spatial autocorrelograms to determine whether model residuals showed spatial dependence. We used the midpoint between the locations of pair members as coordinate references in spatial autocorrelograms (Vilela & Villalobos, 2015). The autocorrelograms show that our models do not suffer from spatial dependence in the residuals (Figure S7).

In the main text, we focus on the results in which the potential for similarity owing to transmission from other close-by societies is computed using, for each society, its five closest neighbours with D-PLACE data. We excluded imbalanced models (the percentage of pairs that show cultural sharing was more than 90% or less than 10%), as well as models that did not converge, or had fewer than 50 datapoints. In total, this analysis included 54 cultural traits (listed in Table S4a). Significance levels were adjusted for false discovery rates to account for multiple independent comparisons (Benjamini & Yekutieli, 2001), using the function p.adjust() in R. Ninety per cent of models show areas under the curve (AUC) higher than 0.8 (the lowest AUC value was 0.73, and five models had AUC values between 0.7 and 0.8, Table S4a), as estimated by the function roc() in the R package {pROC} (Robin et al., 2011).

### Geographic hypothesis: Eurasia's East-West orientation translated into weaker environmental barriers to cultural transmission

#### Quantifying the strength of environmental barriers within corridors of agriculture transmission

To formally test the geographic premise that Eurasia's East–West orientation translated into weaker environmental barriers to cultural transmission, we use a critical test case and arguably the most prominent example in Diamond's (1977) book: the spread of agriculture. Specifically, the axis of orientation hypothesis predicts that the magnitude of ecological barriers to cultural spread should be smaller along potential routes of transmission of agriculture in Eurasia than in other regions of the planet. We used the geographic ranges of 17 known areas of independent origin of domestication of plants and animals as standardised points of reference (Larson et al., 2014). Out of the 19 origin areas proposed in Larson et al. (2014) we excluded New Guinea and Japan, as we are focused here on cultural transmission among continental societies. Following Kavanagh et al. (2018), we considered the Northwestern and Northern Lowlands of South America as one area of domestication origin. We selected societies that are located within these areas based on their geographic positions in Glottolog (Hammarström et al., 2019). As in our prior analyses, we quantify the ecological costs to cultural transmission for pairs of societies. That is, we evaluated environmental barriers between societies located within the areas of independent agricultural origin and each of their closest 100 neighbours. To investigate the potential for long-range transmission, we paired societies within the agricultural points of origin with their closest 100 neighbours located at least 2500 km away (Figure S8 shows the location of societies in each area of agricultural origin, as well as the location of their closest 100 neighbours and closest 100 neighbours located at least 2500 km away). This long-range threshold was chosen in order to investigate the broad-scale cultural transmission examples mentioned in Diamond's thesis, such as from the Fertile Crescent to Southern and Central Europe or to North and East Africa, or from Mesoamerica to Southern North America and Equatorial South America; Figure S8). Therefore, we estimated environmental barriers at both small and large spatial scales within corridors of early agriculture transmission across the world.

For each pair of societies in these analyses we compute the dissimilarity in temperature harshness and aridity index at the locations of the two societies in a pair, accumulated cost and length of the paths of least environmental resistance, accumulated cost and length of the path minimising topographic costs and geodesic distance (Figure 1b and Figure S1b, the green ‘Raw variables’ box). Then, for each focal society (i.e. a society in an agricultural point of origin), we average each of these metrics among its closest 100 neighbours (for short-distance analyses), and among its closes 100 neighbours located at least 2500 km away (for long-distance analyses). Thus, each starting location contributes a single data point in the short- and long-distance analyses, respectively (detailed in Figure S9).

#### Comparing continental differences in ecologically induced costs to agricultural spread

For each society in an agricultural point of origin we have thus quantified the potential costs to cultural transmission from the society's location to its neighbours: dissimilarity in temperature harshness and aridity regimes, cost accumulated along the temperature harshness and aridity index least-cost paths, the lengths of temperature harshness and aridity index least-cost paths, and costs related to human movement (based on elevation and geodesic distances; Figure 1b and Figure S1b, ‘Raw variables’ green box). As we have noted in our ecological test for Diamond's hypothesis, these metrics are highly correlated (Figure S10). We thus follow the same approach and reduce dimensionality in the data by running two separate varimax-rotated PCA analyses: one for close and one for long-range nodes, where a society located in a domestication origin area is the unit of analysis. The close-range PCA gave us three estimates of environmental barriers to cultural spread: (i) a component capturing variation in the topographic travel costs, geodesic distance and climate-related least-cost paths (close-range PC1, or crPC1); (ii) aridity turnover (crPC2); and (iii) temperature turnover (crPC3). As with the first set of analyses, ‘turnover’ here captures environmental dissimilarity at societies’ locations and the accumulated cost across the least-cost path. The long-range PCA gave us four estimates of environmental barriers: (i) temperature turnover (long-range PC1, or lrPC1); (ii) a component capturing the lengths of climate-related least-cost paths (lrPC2); (iii) aridity turnover (lrPC3); and (iv) a component capturing topographic travel costs and geodesic distance (lrPC4, Figure 1b and Figure S1b, the burgundy ‘Variables reduced by PCA’ box). Table S3 shows how each raw variable respectively associated with each close-range and long-range component. We used these principal components in separate linear models: we regressed each PC against a categorical variable with levels represented by 16 areas of domestication origin around the globe (we exclude South India from the analyses, as we only have coordinates for one society in this area of agricultural origin; Figure 1b and Figure S1b, the blue box). We thus ask: are there substantial differences in the mean of each environmental barrier metric (denoted by the values of each principal component) between the areas of agricultural origin? We ran variance-weighted linear models to account for heterogeneity in variance across the levels of the categorical variable. Moran's I tests revealed that models can suffer from spatial autocorrelation in the residuals (Figure S11). We hence accounted for the spatial dependence by using the function SpatialFiltering() in the R package {spatialreg} to select eigenvectors in a semi-parametric spatial filtering approach (Tiefelsdorf & Griffith, 2007). Eigenvectors were introduced as covariates in the models, and accordingly, Moran's I test on the new models showed few spatial dependencies (Figure S11). If Diamond's geographic premise is supported by the data, we expect to consistently see smaller means of environmental barriers (i.e. principal component values) within Eurasian corridors of agricultural spread compared with other regions of our world.

#### Comparing continental differences in ecologically induced costs to agricultural spread

#### using reconstructed climatic conditions at 12, 8 and 4 kya

Because the exact timeline of the spread of agriculture is relatively unknown in many regions of the world, we repeated the analyses in this section using climate reconstructions from different time periods. We obtained values for the reconstructed annual mean and variation of temperature, as well as mean and coefficient of variation of precipitation conditions around the globe at 12, 8 and 4 kya (thousand years ago) from Kavanagh et al. (2018). We choose these timestamps to cover the entire timeframe in which agriculture presumably originated and spread in different regions of the planet (Diamond & Bellwood, 2003; Larson et al., 2014). For each of these time points, we (i) ran a global PCA at a 0.5 × 0.5° resolution, to obtain two axes that capture global variation in temperature harshness and aridity index and (ii) used the two environmental axes computed in (i) to estimate the following environmental barriers to cultural spread within the close- and long-range neighbouring societies located within areas of agricultural origin: dissimilarity in temperature and aridity conditions at the locations of societies, the accumulated cost and length of the least-cost path given a landscape of costs determined by temperature and aridity heterogeneity. The values for the geodesic distance and travel costs (determined by elevation) between societies were not recomputed, as they do not depend on reconstructed temperature harshness and aridity index values. We then repeated the analyses quantifying and comparing differences in the strength of environmental barriers between areas of agricultural origin in order to identify if – at any of the time points considered (12, 8 and 4kya) – Eurasian areas showed consistent ecological advantages over other areas of the world (Table S7). Thus, this section of our analyses broadly asks whether Eurasia exhibits ecological advantages for the transmission of culture either presently or in earlier time points that are relevant to the spread of agriculture.

## Results

### Environmental differences hinder cultural sharing

**Figure 3.**
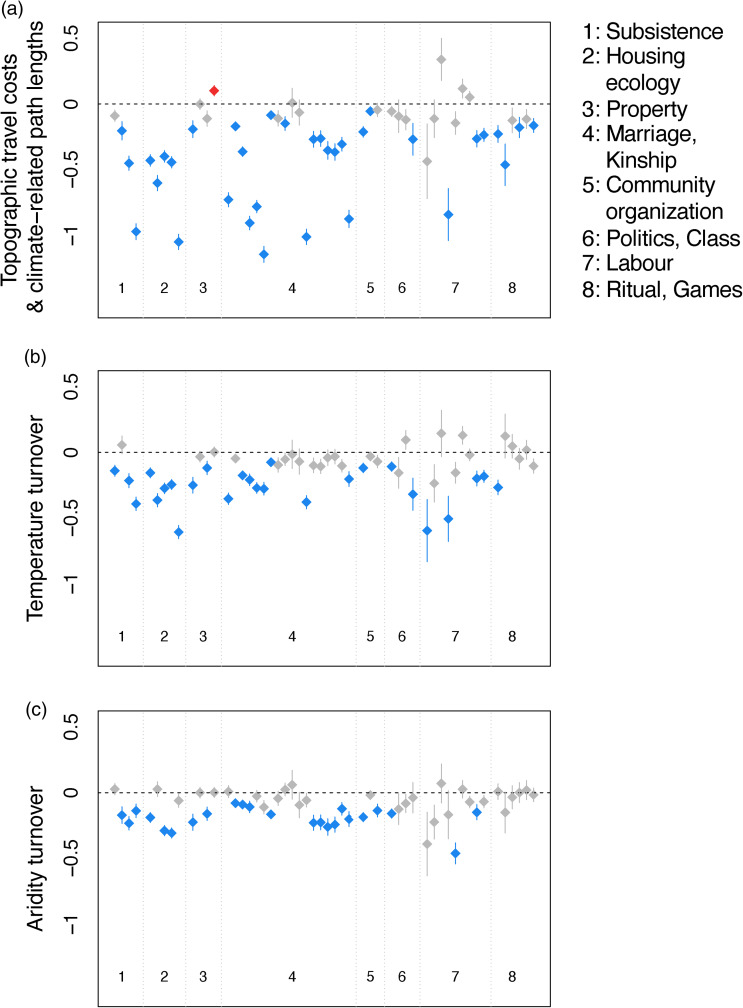
Estimated effects of principal components representing environmental and travel costs to cultural similarity. Each point denotes an independent model. The points’ values represent the estimated effect of the principal component associated with the environmental barriers listed on the *y*-axis label; arrows denote the size of standard errors. Models are binned into broad cultural trait categories (1–8) according to their corresponding response variables. If environmental dissimilarities are associated with a lowered potential for cultural similarity, we expect to see negative values for estimated effects. Non-significant relationships are depicted in grey, whereas significant effects are shown in red (positive) and blue (negative). The *p*-values are adjusted for false discovery rates.

We find that higher costs related to human movement, as well as temperature and aridity turnover show predominantly negative coefficients in our models (Figure 3). That is, travel and environmental barriers to cultural transmission decrease the odds of cultural similarity between societies. Our results, however, also show variation in the strength of these effects among cultural traits and among cost metrics. Costs associated with human movement show higher magnitude coefficients in comparison to temperature and aridity turnover. Further, the negative effects of human movement costs seem consistent among trait categories, with the exceptions of traits related to labour (i.e. specialisation by sex for various activities, Figure 3a). We see that temperature and aridity turnover show mostly significant negative coefficients for traits associated with subsistence, housing ecology, property and community organisation (for aridity turnover). Many traits related to kinship structure also show significant negative relationships with temperature and aridity turnover. Cultural similarity in terms of agriculture intensity does not show a significant negative association with aridity barriers, and major crop type also shows no negative association with temperature costs. Similarity in the predominant type of domestic animal a society uses and dominant subsistence economy shows significant negative associations with all our cost metrics. As expected, we found that trait sharing is generally more likely among close relatives and in situations where the potential for similarity owing to cultural transmission from other nearby neighbours is high (Table S4a). We also observe predominantly negative coefficients of costs associated with human movement and environmental turnover in our sensitivity analysis – i.e. when we account for the potential of cultural transmission between pairs of societies from other close-by societies using the 10 closest neighbours regardless of whether or not these had cultural data in D-PLACE (Figure S12, Table S4b). Further, running the analyses on a randomly drawn set of language posterior trees returned similar results to the main text analyses (that use a maximum clade credibility tree, Data S3). Thus, our results generally support Diamond's core assumption – i.e. that environmental similarities facilitate the transmission of cultural traits (Diamond, 1997). However, our findings also show a more nuanced picture in which the strength of travel and environmental barriers can vary among trait categories.

### Environmental barriers to cultural transmission are not weaker in Eurasia

**Figure 4.**
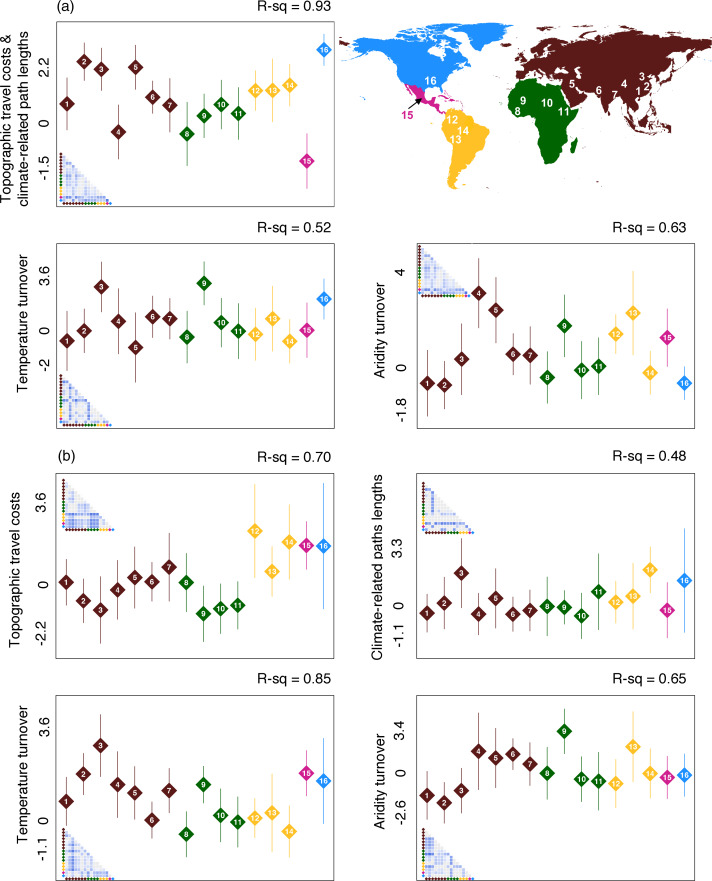
Mean and standard deviation of principal components representing environmental costs to cultural transmission in 16 known areas of domestication origin (1, South Tropical China; 2, Lower-Middle Yangtze; 3, Chinese loess plateau; 4, West Yunan and East Tibet; 5, Fertile Crescent; 6, Sava West India; 7, Ganges of East India; 8, West Africa; 9, West African Savannah; 10, Sudanic Savannah; 11, Ethiopian plateau; 12, Northern Lowlands of South America; 13, Central/Southern Andes; 14, Southwest Amazon; 15, Mesoamerica; 16, East North America). Each graph represents a separate model, where the response variable is a principal component capturing variation in the environmental barriers listed on the *y*-axis label. Colours highlight different major landmasses (see inset map). Panels show the comparison of environmental barriers at (a) close- and (b) long-range spatial scales. Inset matrices show pairwise Tukey's honest significant differences, with stronger blue hues depicting higher magnitudes of difference. Non-significant differences are shown in grey. Following Diamond's hypothesis, we expect smaller means for environmental costs to cultural transmission in Eurasian areas compared with other areas of the globe.

Our analyses uncovered significant variation within continents in the magnitude of ecological barriers to cultural transmission (Figure 4 and Figure S13), a finding that is consistent with the critique that Diamond's axis of orientation hypothesis is potentially reductionist and overreaching (Blaut, 1999). More importantly, our results indicate that areas within Eurasia do not consistently rank lower than those in other continents across any cost type (in Figure 4, Eurasian areas do not consistently exhibit lower environmental barriers to cultural transmission than those in other continents; Table S5). Our sensitivity analyses revealed that at none of the time points considered (12, 8 and 4 kya; Figure S14 and Tables S6 and S7) did Eurasia have any clear ecological advantages over other regions. Thus, we find no support for Diamond's claims during the timeframe in which most societies around the world transitioned into an agriculturalist mode of subsistence.

## Discussion

In this paper we have provided a data-driven approach to test the core ecological and geographic premises of the axis of orientation hypothesis, i.e. the idea that Eurasia's East–West axis of orientation enabled a faster spread of cultural innovations and contributed to the region's early development. Specifically, we have shown that environmental differences in temperature and aridity are associated with decreased odds of cultural similarity, supporting the premise that ecology can influence the potential for cultural transmission. Nevertheless, our results also uncover a complex picture, in which ecological influences on cultural similarity can vary between different cultural traits, showcasing the complexities of cultural transmission and the potential weakness of single-factor explanations. Further, our findings show that there is no convincing evidence that the geography of Eurasia imposed smaller ecological barriers to the early transmission of agriculture than those of other continents around the globe. Thus, while we found support for the role of ecology in shaping the transmission of culture, it does not appear that such a mechanism actively biased the spread of cultural innovations in the Eurasian continent, at least as it relates to the spread of early agriculture.

The underlying idea of the axis of orientation hypothesis is that fast cultural transmission may have facilitated the development of large, dense, sedentary and stratified societies. Our analyses help test this hypothesis by covering a wide variety of cultural traits, including some that directly relate to social development (e.g. dominant mode of subsistence, domestic animal and major crop type, agriculture intensity, settlement patterns, political complexity traits – jurisdictional hierarchy of local and beyond local community, political integration or class differentiation). We found that in general, costs of human travel had larger effects than those of aridity or temperature turnover, indicating that mobility is likely to affect cultural transmission more strongly than the environment. However, we note that these effects may not be solely attributed to mobility because our metrics of costs to human travel, particularly geographic distance, are likely to capture the effects of other unmeasured promoters of cultural similarity including the likelihood of direct contact between cultural groups. Given the clear links between environment and human subsistence or housing, it is not surprising that the transmission of these important cultural traits generally showed significant associations with geographical and ecological barriers. Similarly, it is not surprising that traits related to sexual segregation of labour, games and rituals were less consistently affected by environmental barriers because such traits tend to be more idiosyncratic and culturally driven. Importantly, in line with Diamond's thinking, we found evidence of some ecological biases in the transmission of domesticated animals and subsistence types, agriculture intensity, and major crop type. Nevertheless, we note that sharing the same major crop type was not significantly associated with temperature turnover, which is consistent with earlier critiques that domesticated plants can often adapt to new conditions, particularly if humans assist them by modifying their immediate surroundings (Blaut, 1999; Fuller & Qin, 2009).

We also note that while our findings generally support Diamond's expectation of environmental barriers to cultural transmission, the generality of this effect must be treated with care. For example, we note that our ability to detect significant effects could in some cases depend on coding schemes or on the active transmission of other cultural traits. Coding schemes for cultural traits are a particularly complex issue (Slingerland et al., 2020). Some traits are coded into finer categories than others (e.g. marital residence with kin has 12 different categories, whereas the characteristics of moralising high gods has only four). Additionally, the quality and quantity of sources, as well as the focal years of study, vary among societies and traits, meaning that different analyses may be subject to different levels of statistical noise. As for the possibility of correlated transmission of cultural traits, we note that although our study evaluated each trait independently, it is conceivable that some cultural traits are transmitted in packages, meaning that some of the effects we report could be driven by the active transmission of only a few key traits. For example, if traits related to plant and animal domestication tend to be adopted with other kinds of traits, then those other traits would exhibit environmental biases even if they do not have an actual bias by themselves (as Diamond himself argued).

Our second set of analyses compared the distribution of ecological barriers to cultural transmission between 16 important areas of the globe: centres of agricultural origin. While the first set of analyses tests a key ecological assumption in Diamond's theory (ecological biases in cultural spread), it is this second set of analyses that tackles (and casts doubt upon) Diamond's overarching message: Eurasia benefitted from a more homogenous environment along its major corridors of cultural transmission. One of his most prominent arguments in this regard was that agriculture spread very rapidly out of the Fertile Crescent. Contrary to that view, we found that this particular region can actually experience stronger environmental barriers within the corridors of agricultural spread than those observed in other centres of agricultural origin. This trend is particularly observable when considering aridity turnover at both close- and long-range, as well as close-range topographic costs. The likely reason for this discrepancy is that even though the Fertile Crescent has access to a larger area of contiguous landmass at similar latitudes than many of its peers (Diamond's main argument), it is nevertheless more ecologically atypical for its surroundings. Most notably, the Fertile Crescent is nourished by rivers but surrounded by large deserts, and these strong gradients in access to water are further compounded by nearby changes in prevailing winds and elevation (Figure S15). These findings remind us that dramatic changes in habitat and climate can occur even within small spatial scales. In contrast to the Fertile Crescent (and other Eurasian areas), South Tropical China, the Lower-Middle Yangtze and the Chinese plateau stand out as having low aridity related costs compared with most centres of agriculture origin. South Tropical China also shows low to mid-range temperature turnover values. Both aridity and temperature regimes would have been important for the spread of rice agriculture (d'Alpoim Guedes & Butler, 2014; Gutaker et al., 2020), although archaeological evidence shows that human-directed water management systems were developed early and were part of the success of rice as a crop in these regions (Fuller & Qin, 2009). Thus, humans were working towards hijacking the conditions that were set for their crops by the local environment, a finding that strengthens the need to consider cultural factors when understanding crop domestication. Our results show that close-range topographic travel costs are not trivial in South Tropical China, and the Lower-Middle Yangtze and the Chinese plateau show one of the highest barrier levels along this axis. Thus overall, we do not find a consensus of universal low environmental barriers to cultural spread in Eurasian areas, as hypothesised based on Eurasia's East–West dominant continental axis.

For the second set of analyses, the choice of which societies to include was not restricted to the Ethnographic Atlas, rather it was based only on the Glottolog (Hammarström et al., 2019), thus alleviating the Ethnographic Atlas sampling bias against large-scale cultural groups. Nonetheless, cultural range size can be positively linked with latitude (Gavin & Stepp, 2014), meaning that Eurasian societies could be biased towards larger group sizes in our sample. Using point coordinates to identify the close-range and long-range 100 neighbours for each society in an agricultural point of origin could also inflate cost metrics for wide-ranged societies. Together, these could result in biases for Eurasian societies towards high costs, particularly when metrics are based on geographic distances. However, the PCA applied on the raw cost variables isolates geographic distances from environmental turnover, which could alleviate some of the potential bias against large-scale temperate societies by allowing us to independently assess the effect of ecological barriers versus geographic distances between pairs of societies. We also emphasise that the uneven distribution of distances between societies located in various agricultural centres of origin and their neighbours is not a product of uneven sampling, but rather a product of true unevenness in the settlement and mobility patterns of people in different parts of the world (e.g. environmental constraints enable a higher density of distinct cultural groups close to the tropics and near mountain peaks; Gavin & Stepp, 2014). While our neighbourhoods may cover different radii, such differences probably reflect real differences in what is probably accessible to the people in different focal societies (societies in centres of agricultural origin). That is, our approach to investigating the potential for cultural transmission (loosely defined as inclusive of both the demic and non-demic transmission of cultural innovations) acknowledges that the typical distance covered by the out-group people that a society interacts with varies across the world due to constraints on human movement (e.g. topography) and human settlement patterns (e.g. natural resources).

The axis of orientation hypothesis is primarily framed around cultural diffusion. However, it is likely that the spread of culture is strongly tied to the movement of peoples and the expansion of populations as well. For example, the East–West spread of empires (Turchin et al., 2006), and the lower language turnover within latitudinal bands (Laitin et al., 2012) support the notion that cultural spread may depend, at least in part, on demic diffusion. Our finding that both the costs of human travel and ecological heterogeneity affect cultural similarity also support this idea.

It is important to note that the axis of orientation hypothesis is only one of several non-exclusive environmental hypotheses that Diamond himself proposed as potential explanations for the way in which history unfolded on different continental regions. Among these ideas, two of the least controversial relate to opportunity and cost. For example, Diamond noted that the Americas and Africa may have simply offered fewer domesticable species than Eurasia, highlighting the fact that Eurasians were able to domesticate 13 species of large animals whereas South Americans were able to domesticate only one (alpaca/llama). Diamond also viewed disease as another potentially important contributor to historical asymmetries. Specifically, he argued that environmental similarity could have favoured the rapid spread of parasites and driven a quicker homogenisation of zoonoses in the Eurasian continent. Thus, in his view, Eurasians may have more quickly become less susceptible to an extensive list of pathogens and may have therefore been able to subsequently progress at a faster rate than humans in other continental regions. In addition to these alternatives, we note that historical contingency and cultural factors can also potentially outsize the effect of environmental biases on human history. For example, the differential timing of human colonisation in different continents may have simply afforded South Americans less time to develop large, dense, sedentary and stratified societies in comparison to Eurasians.

In conclusion, here we leveraged a wealth of existing data on climate, geography and culture to quantitatively test an influential idea on the history of human development. By operationalising the axis of orientation hypothesis, we have shown that although Diamond's intuition that ecological similarity facilitates cultural transmission is probably correct, these ecological effects are unlikely to be as influential as Diamond intuited. Moreso, we found no support for his argument that key areas of Eurasia are more ecologically homogeneous than comparable sites in other regions of the world. Our findings point out that latitude, like genetics and ecology, is not destiny (Blaut, 1999). We echo earlier concerns about the perils of single factor explanations and suggest that chance, and perhaps factors that promoted colonial empires, need to be more seriously considered as potentially important drivers of human inequality.

## Supporting information

Chira et al. supplementary material Chira et al. supplementary material

## Data Availability

The code for performing the analyses can be found in a public repository on GITHub (https://github.com/angela-mc/AxisOrientationHypothesis). Other data files have been uploaded on ZENODO: https://zenodo.org/records/10401227?token=eyJhbGciOiJIUzUxMiJ9.eyJpZCI6IjU3ZmFlN2RmLThiOGItNDkxZS04Y2NlLTczMzU3MDA4MDA3NSIsImRhdGEiOnt9LCJyYW5kb20iOiIxNzc2MzZlNmE5ZDMwM2VhODA5YTk3NjI0NzI3NDRlYSJ9.as8dxnbiqCSSmu7UolqGSNT9f7cSybRk6nDYtdNVQ5GwaUzdwIkj-gv8GWKpmUJExrlVnjuIeWBPTQP_jux3rg. Cultural data are available in the open-access D-PLACE database accessible at https://d-place.org.

## Supplementary material

To view supplementary material for this article, please visit https://doi.org/10.1017/ehs.2023.34
