# Supplementary material for: Geography is not destiny: A quantitative test of Diamond's axis of orientation hypothesis
Source: Evol Hum Sci. 2024 Jan 4;6:e5. doi: 10.1017/ehs.2023.34 (PMC10808877; doi:10.1017/ehs.2023.34)
Supplement: Chira et al. supplementary material [file S2513843X23000348sup001.pdf]

a) Ecological hypothesis

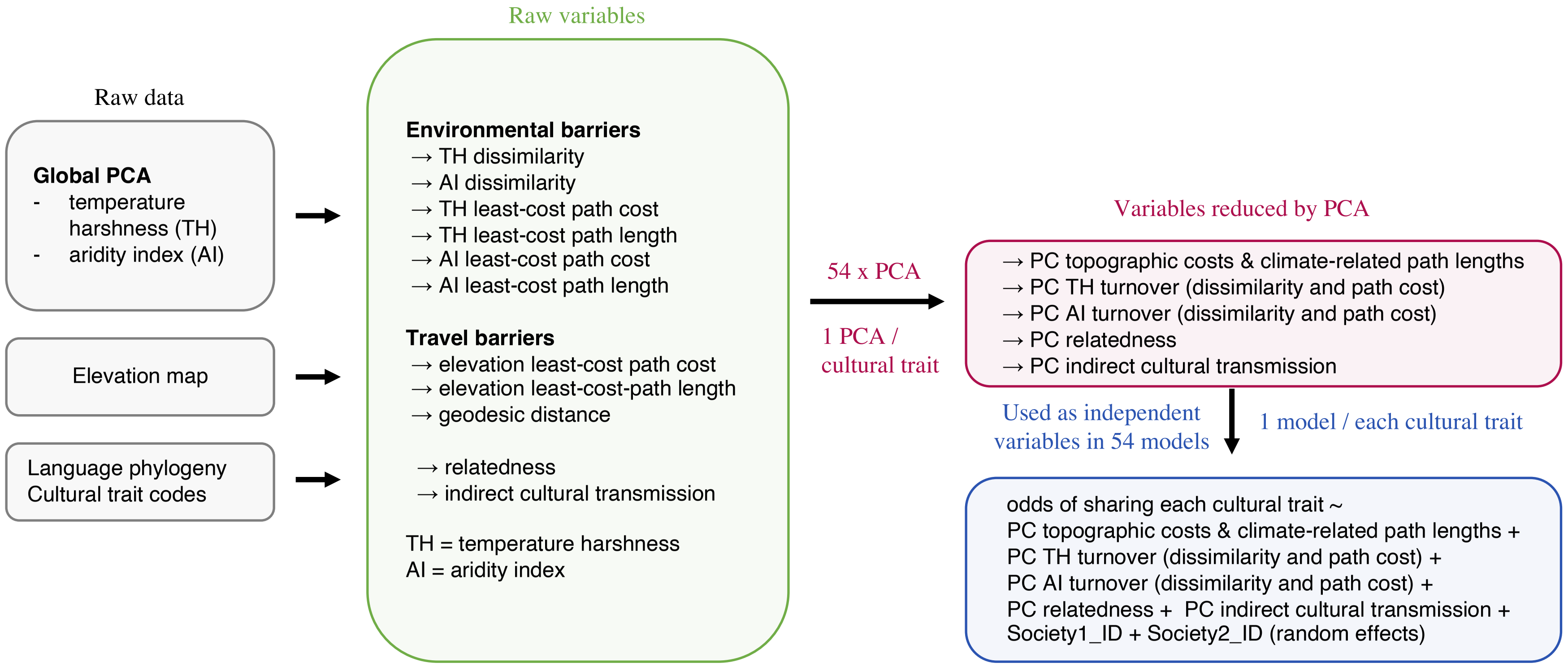

Figure S1a.
(a) General workflow for testing the ecological premise of Diamond's hypothesis. A global PCA at 0.5° x 0.5° resolution is first used to derive variation in temperature harshness (TH) and aridity index (AI) around the world. These climatic variables are then used to compute environmental barriers to cultural spread between pairs of societies. Environmental and travel barriers (obtained from data on global elevation levels), along with metrics of relatedness and cultural transmission from outside the pari (i.e., 'Raw variables') are ran through PCAs (one for each cultural trait) to extract five principal components (PCs, i.e., 'Variables reduced by PCA'). These PCs are then used as independent variables in 54 separate models predicting the odds of sharing key cultural practices between societies in pairs. The identities of societies in pairs were also added in models as random effects.

b) Geographic hypothesis

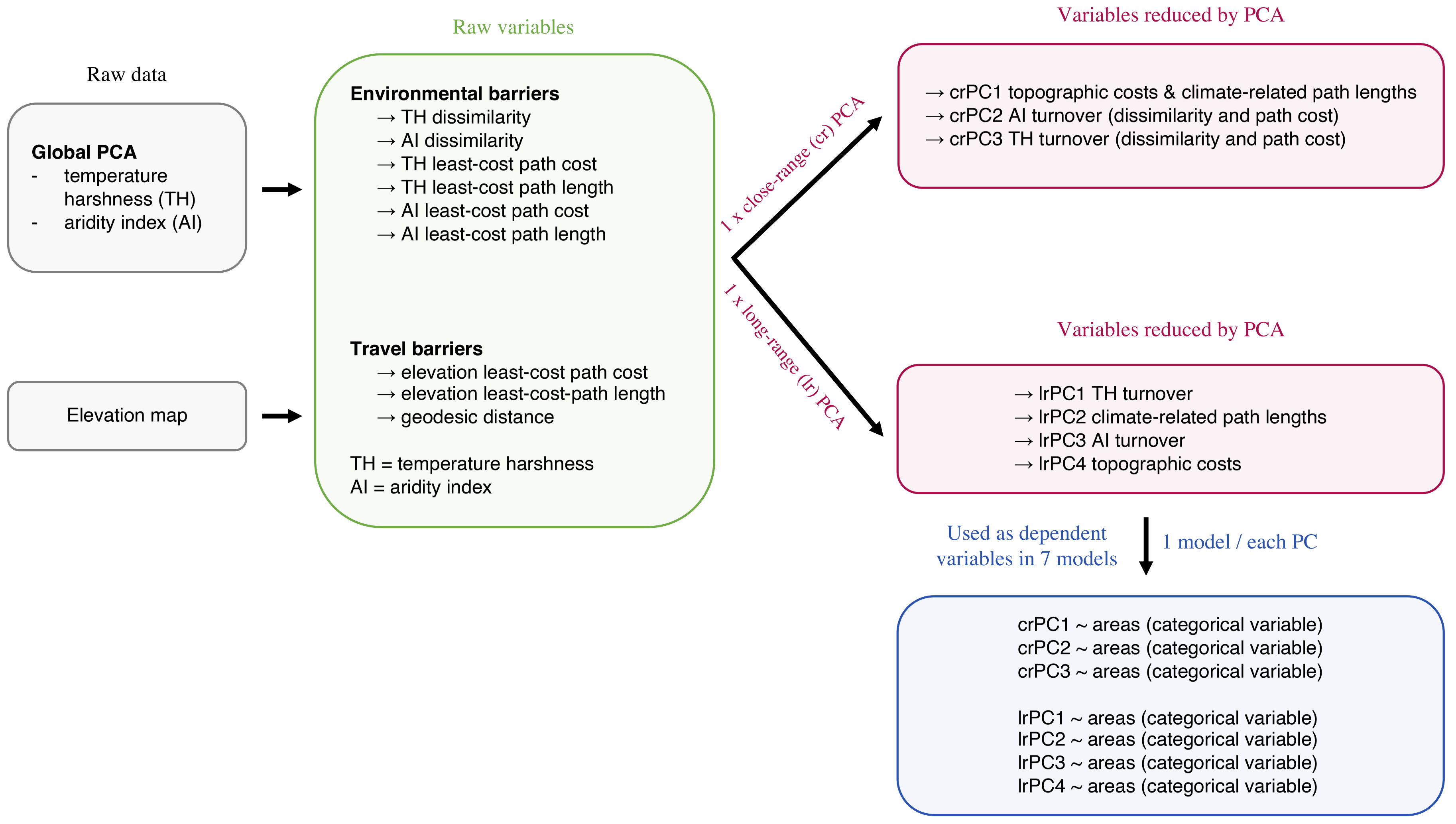

Figure S1b.
(b) General workflow for testing the geographic premise of Diamond's hypothesis. A global PCA at 0.5° x 0.5° resolution is first used to derive variation in temperature harshness (TH) and aridity index (AI) around the world. These climatic variables are then used to compute environmental barriers to cultural spread between pairs of societies. Environmental and travel barriers (i.e., Raw variables, obtained from data on global elevation levels) are ran through two separate PCAs. This way, we cover close- and long- range spatial scales. We then extract three close-range (cr) and four long-range (lr) PCs as estimates of environmental barriers (i.e., 'Variables reduced by PCA) and use them as response variables in seven separate models (i.e., 1 model for each PC). In all models, the independent factor is a categorical variable showing the membership of societies to various areas of agricultural origin.

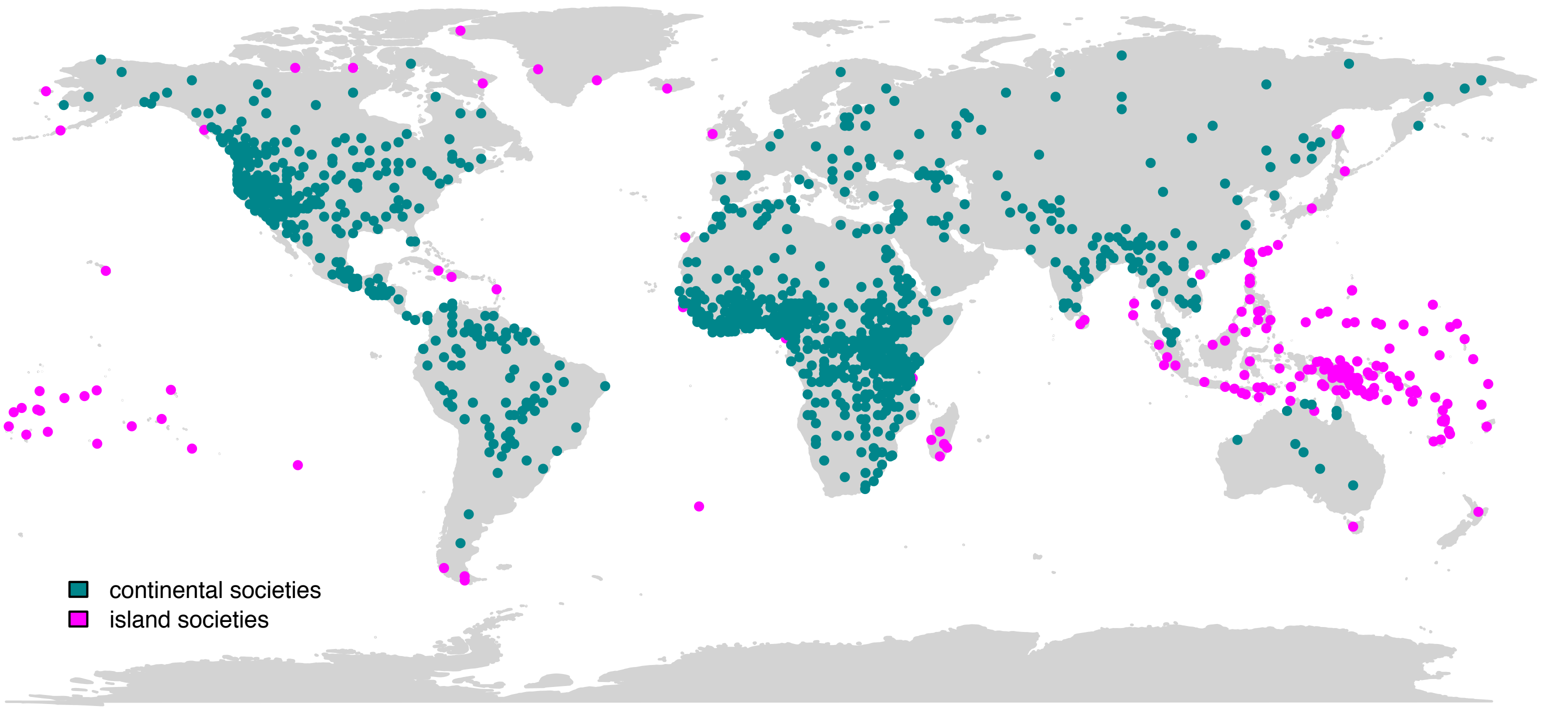

Figure S2.
Distribution of 1,291 societies for which cultural data is recorded in the D-PLACE database. Societies are plotted using the coordinates of their midpoint range, and the 1,094 continental societies used in this paper are shown in turquoise.

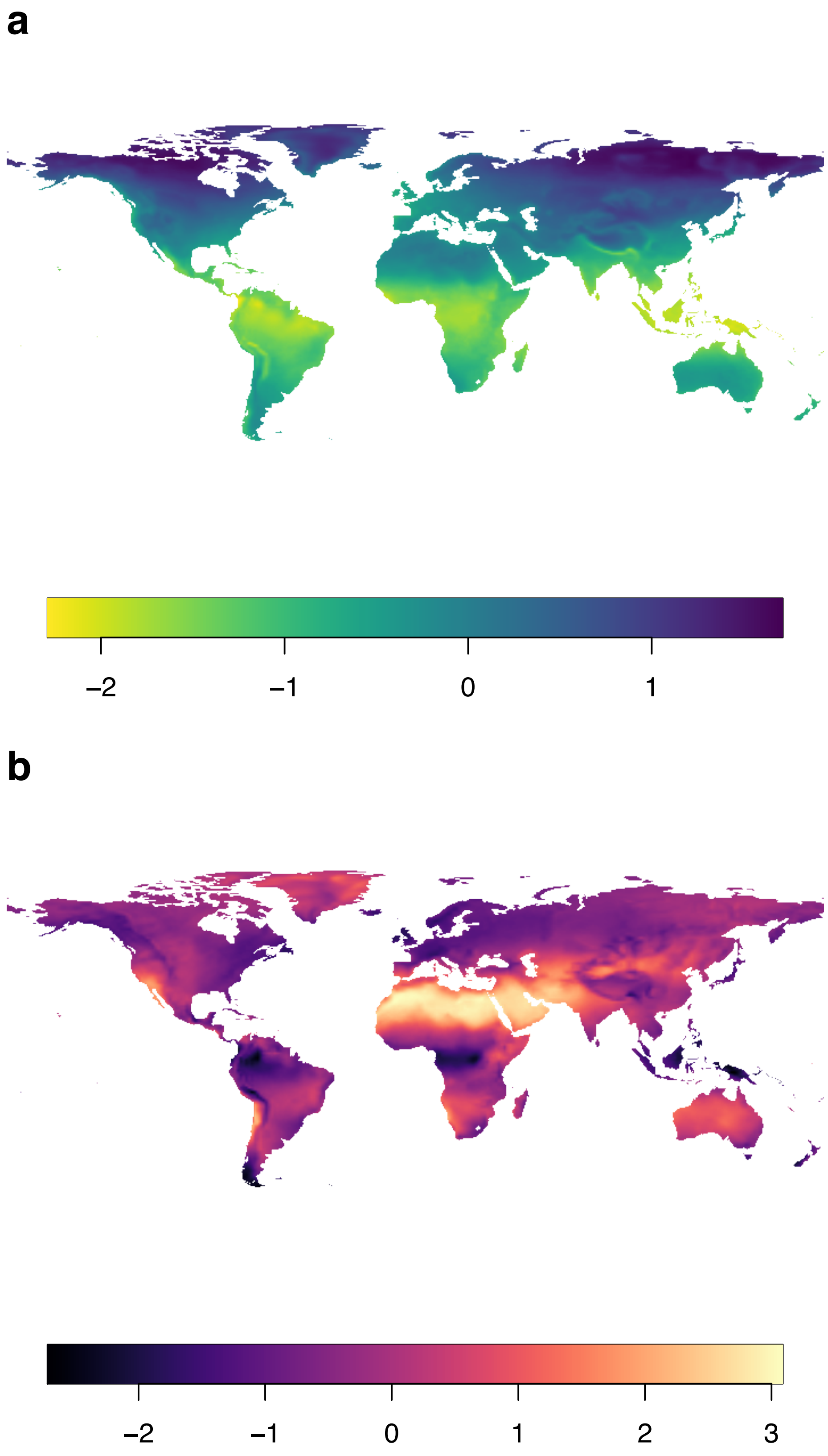

Figure S3.

Maps of temperature harshness (b) and aridity index (b), two principal components obtained from a global PCA at a 0.5° x 0.5° resolution, which includes: annual mean temperature, annual temperature variance, temperature predictability, mean annual precipitation, annual precipitation coefficient of variation, and precipitation predictability. Each map cell is associated with a value for the two components: (a) temperature harshness - a continuum from warm, predictable, and invariable to cold, unpredictable, and variable conditions, and (b) Aridity index - a continuum from humid, predictable, and invariable to dry, unpredictable, and variable conditions.

Figure S4.
Workflow for computing variables of interest for testing the ecological premises of Diamond's thesis.

Society pair: AB
Cultural trait of interest: subsistence mode

a) Determine cultural similarity:
Do '**A**' and '**B**' share the same subsistence mode? Yes/No

b) Determine environmental dissimilarity between the home ranges of **A** and **B**

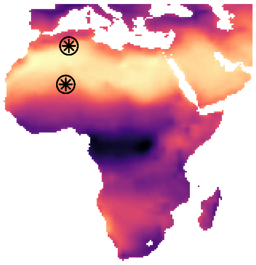

- take aridity index (AI) map
- aridity index dissimilarity:
  abs(AI values at location of **A** – AI values at the location of **B**)

- repeat for temperature harshness map, to obtain temperature regime dissimilarity

c) Determine environmental heterogeneity in terms of aridity index in the intervening space between **A** and **B**
- start with society A and compute least-cost aridity index path **A → B**
  - take aridity index map
  - build a map where costs are proportional to dissimilarities in aridity index from the starting location (here, location of **A**). That is, each cell value across the cost surface is computed as the absolute difference in aridity index between the cell's location and the starting point (here, location of **A**)
  - compute the paths of least resistance using the {gdist} package in R (van Etten, 2017), setting the transitions between cells using a 1/destination cost function, i.e., at each step, the path accumulates the cost of the destination cell. Thus, a higher total cost accumulated across the paths of least environmental resistance indicate high environmental dissimilarities compared to the starting point (here, location of **A**)
  - record the accumulated cost and length of path **A → B**

- repeat for path **B → A**, to obtain accumulated cost and length of path **B → A**

- environmental heterogeneity in terms of aridity index in the intervening space between **A** and **B** is given by: mean (path cost **A → B**, path cost **B → A**), and mean (path length **A → B**, path length **B → A**)

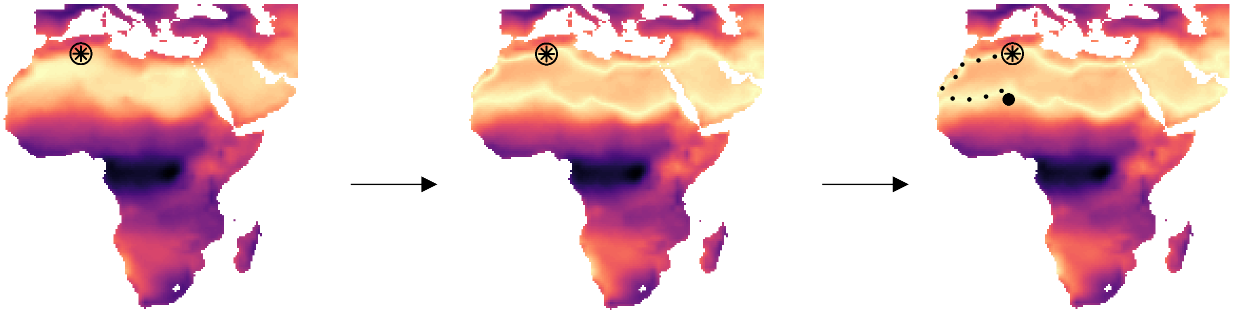

aridity index map — aridity cost map — path of least resistance

Figure S4.
Workflow for computing variables of interest for testing the ecological premises of Diamond's thesis – continuation from previous page.

Society pair: AB
Cultural trait of interest: subsistence mode

d) Determine environmental heterogeneity in terms of temperature regies in the intervening space between **A** and **B** by repeating the above algorithm for the temperature harshness map.

e) Determine least-cost topographic paths between **A** and **B**.
- take elevation map
- use Tobler's hiking function as implemented in the R package {movecost} (Alberti, 2019) to compute the least-cost paths between societies across an elevation terrain (see Figure 1 in the main text for an example).

f) Compute the geodesic distance between societies.

g) Thus, for each pair of societies **AB**, we computed the following (see Figure 1, Figure S1):
- temperature harshness dissimilarity between the locations of **A** and **B**
- aridity harshness dissimilarity between the locations of **A** and **B**
- cost of least-cost-path in terms of temperature harshness
- length of least-cost-path in terms of temperature harshness
- cost of least-cost-path in terms of aridity index
- length of least-cost-path in terms of aridity index
- cost of least-cost-path in terms of human travelling costs (based on elevation)
- length of least-cost-path in terms of human travelling costs (based on elevation)
- geodesic distance

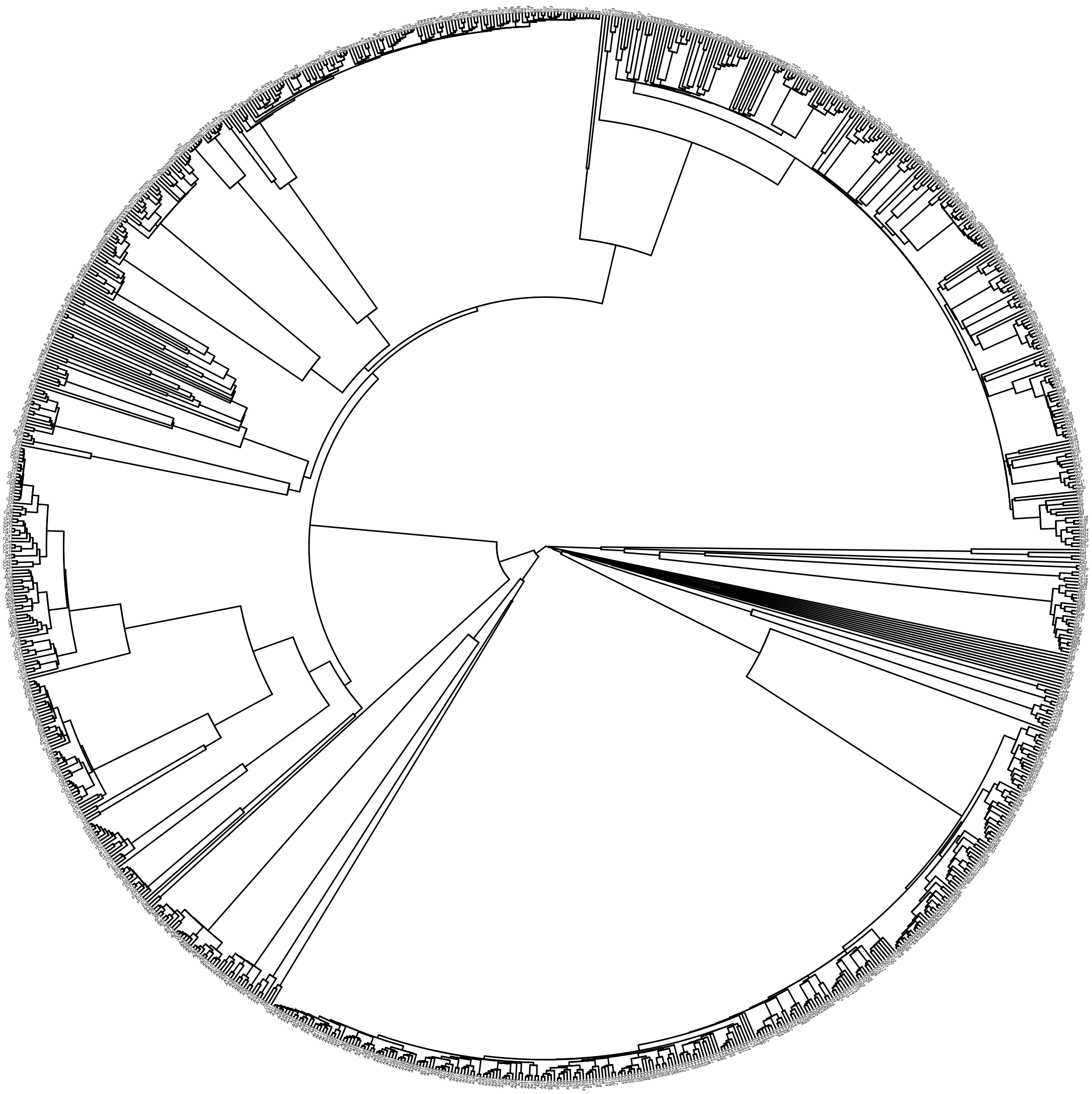

Figure S5.
Phylogeny of languages, pruned for societies in our analyses.

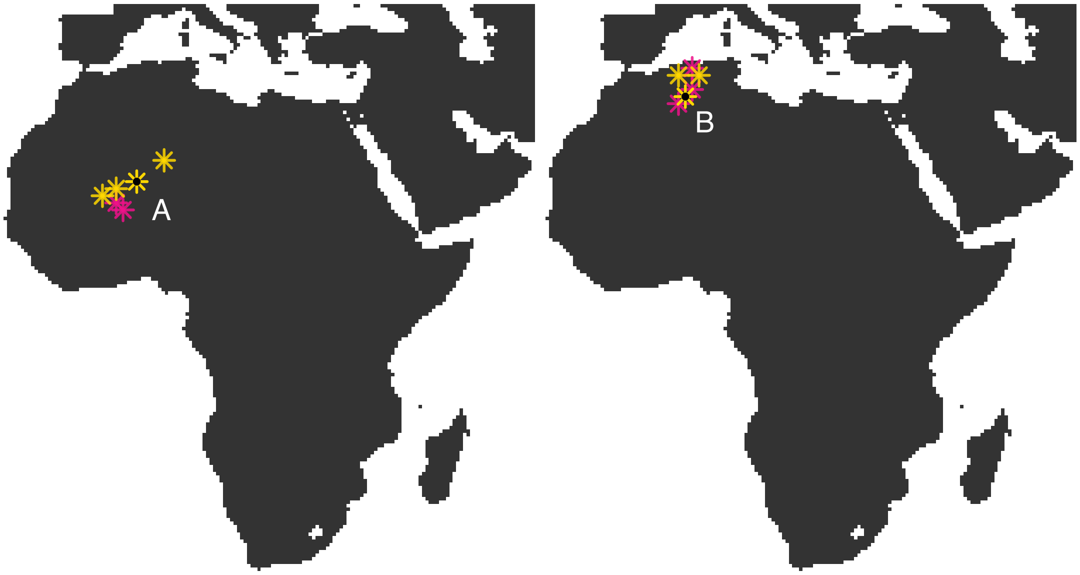

% neighbors of ‘**A**’ with trait of ‘**B**’ (pastoralism) = 3/5

% neighbors of ‘**B**’ with trait of ‘**A**’ (pastoralism) = 2/5

chance of cultural similarity between ‘**AB**’ as a result of
cultural transmission from other nearby neighbours:
mean(3/5,2/5) = 0.5

Figure S6.
Quantifying the possibility of cultural similarity between societies in pairs as a result of cultural transmission from other nearby neighbours. Each point represents the location of a society, coloured according to the dominant mode of subsistence (yellow = pastoralism, pink = agriculture). The two panels show each of the societies in the example pair ‘AB’ (distinguishable from other societies on the map by a central black dot), and their nearest five neighbours with cultural data available in the D-PLACE database.

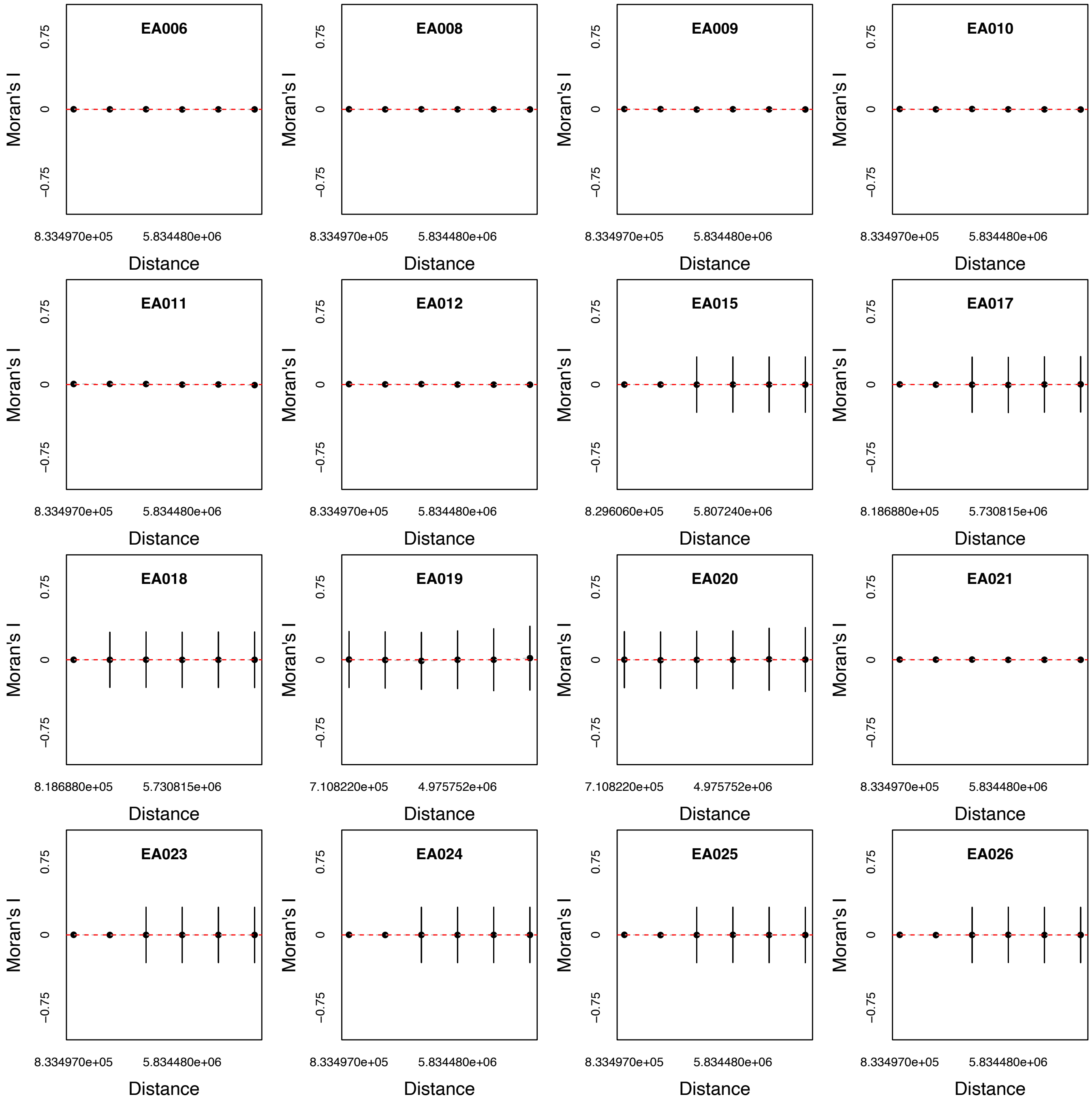

Figure S7.
Moran's I spatial autocorrelograms of residuals corresponding to models presented in Figure 3. Each model is representative for a cultural trait denoted by its D-PLACE variable code. Each point represents a distance category, and circles close to zero indicate no/low levels of spatial autorcorrelation in model residuals. (continues overleaf)

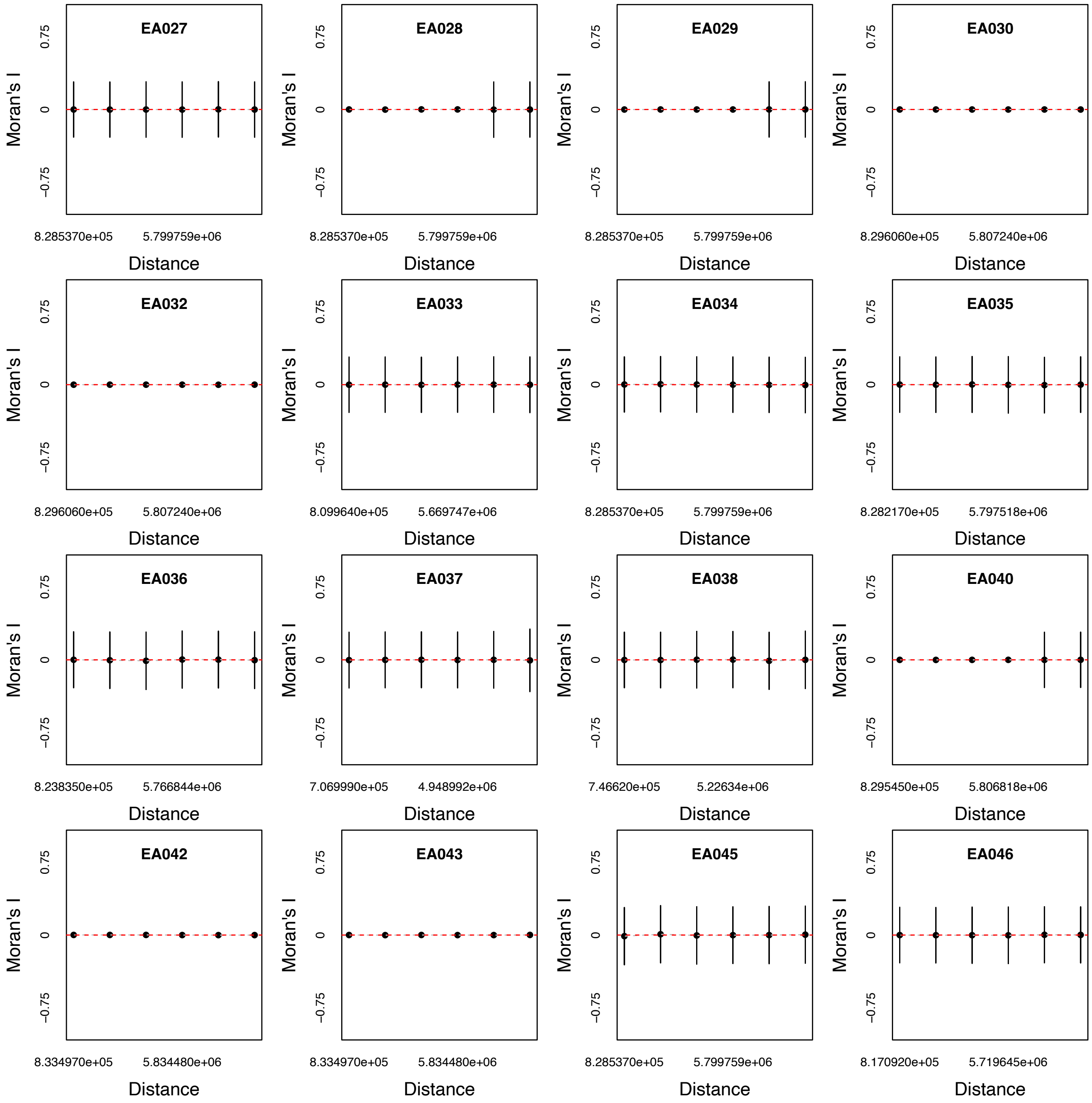
EA027
EA028
EA029
EA030
EA032
EA033
EA034
EA035
EA036
EA037
EA038
EA040
EA042
EA043
EA045
EA046
Moran's I
0.75
0
−0.75
Distance
8.285370e+05
5.799759e+06
8.296060e+05
5.807240e+06
8.099640e+05
5.669747e+06
8.282170e+05
5.797518e+06
8.238350e+05
5.766844e+06
7.069990e+05
4.948992e+06
7.46620e+05
5.22634e+06
8.295450e+05
5.806818e+06
8.334970e+05
5.834480e+06
8.170920e+05
5.719645e+06

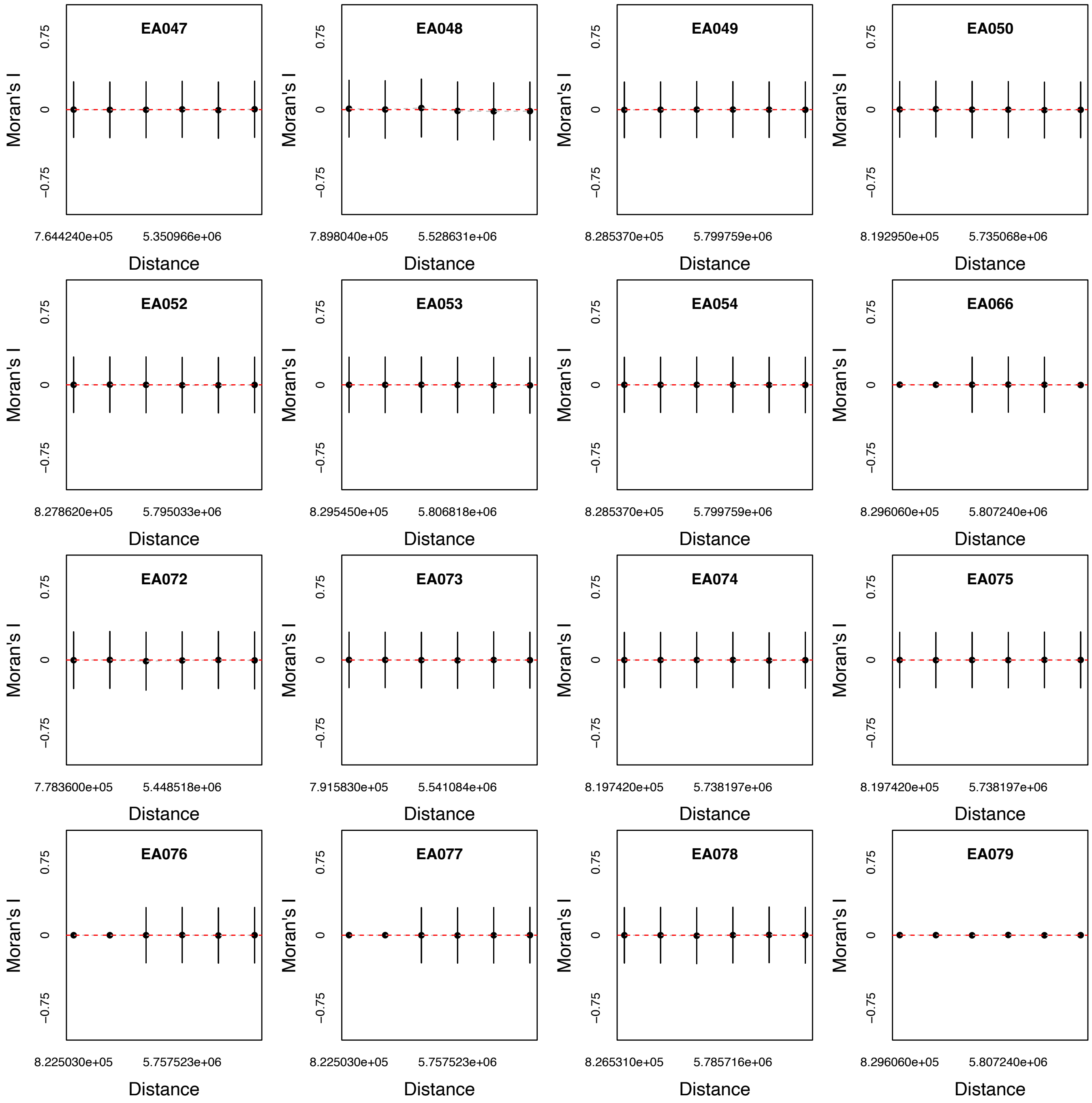
EA047
EA048
EA049
EA050
EA052
EA053
EA054
EA066
EA072
EA073
EA074
EA075
EA076
EA077
EA078
EA079
Moran's I
0.75
0
−0.75
Distance
7.644240e+05
5.350966e+06
7.898040e+05
5.528631e+06
8.285370e+05
5.799759e+06
8.192950e+05
5.735068e+06
8.278620e+05
5.795033e+06
8.295450e+05
5.806818e+06
8.296060e+05
5.807240e+06
7.783600e+05
5.448518e+06
7.915830e+05
5.541084e+06
8.197420e+05
5.738197e+06
8.225030e+05
5.757523e+06
8.265310e+05
5.785716e+06

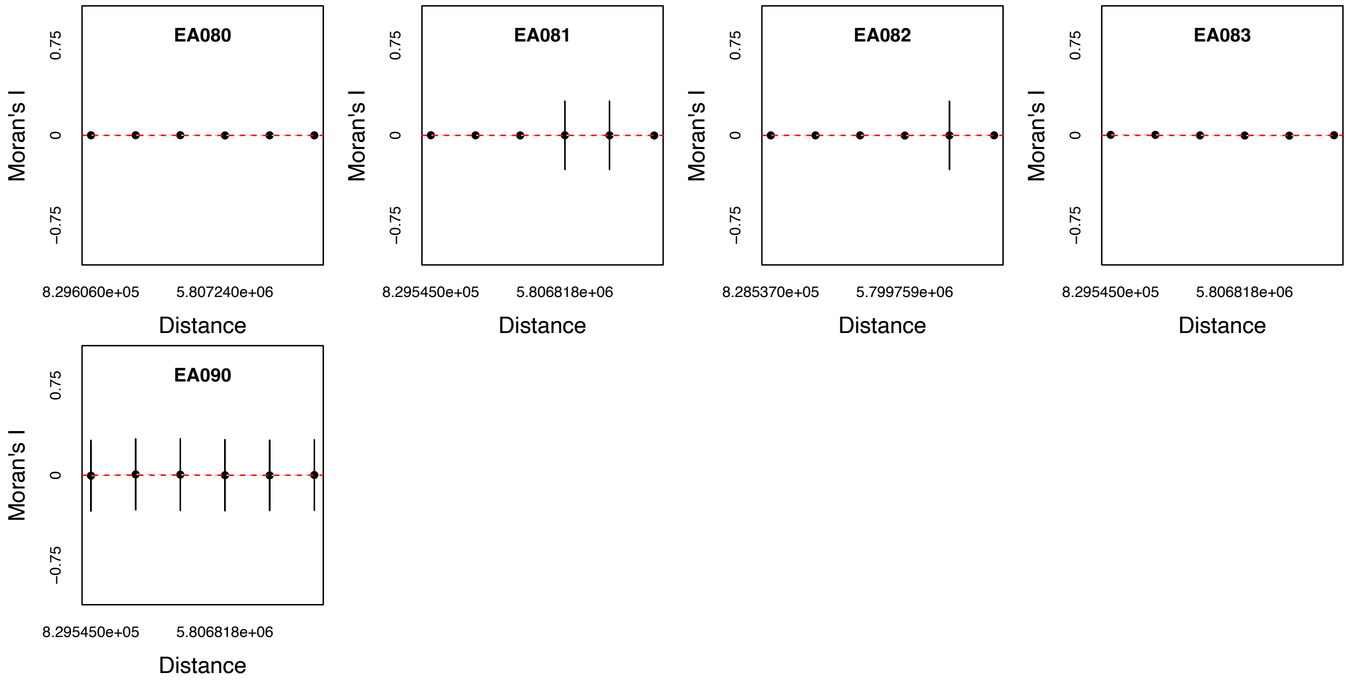

EA080
EA081
EA082
EA083
EA090
Moran's I
0.75
0
−0.75
Distance
8.296060e+05
5.807240e+06
8.295450e+05
5.806818e+06
8.285370e+05
5.799759e+06

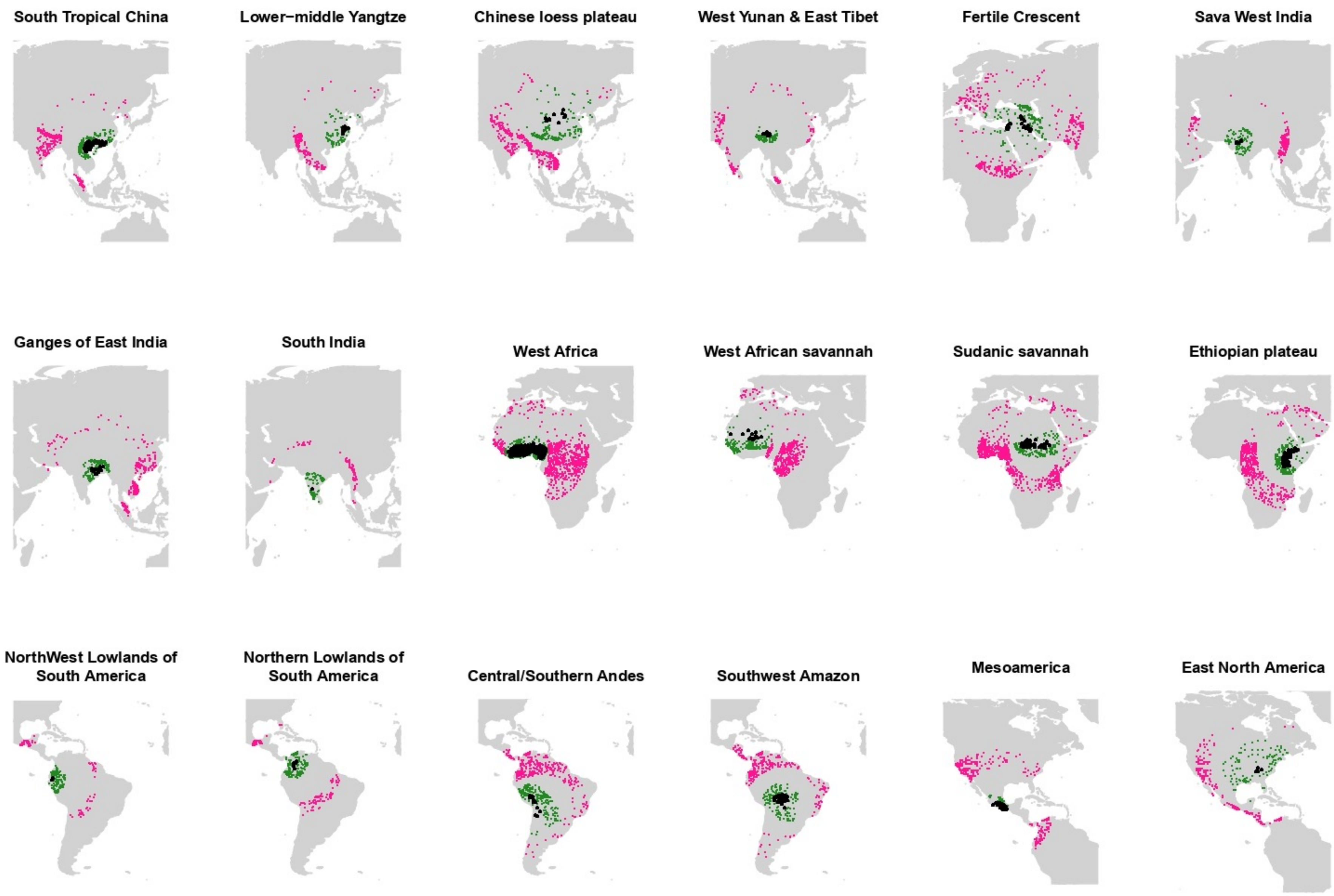

Figure S8.
Distribution of close- and long- range neighbours for societies associated with 17 known areas of independent agricultural origin. Focal societies are coloured in black, whereas their close- (nearest 100) and long-range (nearest 100 societies beyond a radius of 2,500km) neighbours are coloured in green and dark pink, respectively. All maps span 85° longitude and 100° latitude. Neighbouring societies are determined using the geodesic distance. In our analyses, we considered the Northwestern and Northern Lowlands of South America as one area of domestication origin (following Kavanagh et al., 2018). Further, South India was excluded from downstream analyses because only one society is located in this area.

Figure S9.
Workflow for computing variables of interest for testing the geographic premises of Diamond's thesis.

Focal society (located within an agricultural origin centre): A
Example flow for long-distance analyses

a) Determine A's closest 100 neighbours located 2,500 km away: societies B1, B2, …, B100 (labelled B[1-100] in the text below).

b) Determine environmental dissimilarity in terms of temperature harshness between the home ranges of A and the home ranges of societies B[1-100]; average these values to obtain a single value for society A.

c) Repeat b) for aridity index.

d) Determine environmental heterogeneity in terms of temperature harshness in the intervening space between A and societies B[1-100]; average these values to obtain a value for accumulated cost and one value for path length for society A.

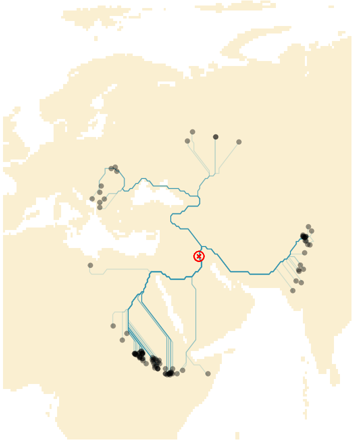

Temperature harshness least-cost paths from the location of a society within the Fertile Crescent (in red) towards its closest 100 neighbours located further than 2,500 km away. The accumulated costs along these paths are averaged to obtain a single value for temperature harshness least-cost path cost for this society. The lengths of these paths are averaged to obtain a single value for temperature harshness least-cost path length for this society.

e) Repeat d) for aridity index.

f) Determine least-cost topographic paths between A and societies B[1-100]; average these values to obtain a a single value for average travel accumulated cost and one value for average path length for society A.

g) Determine the geodesic distance between A and societies B[100]; average these values to obtain a single value for society A.

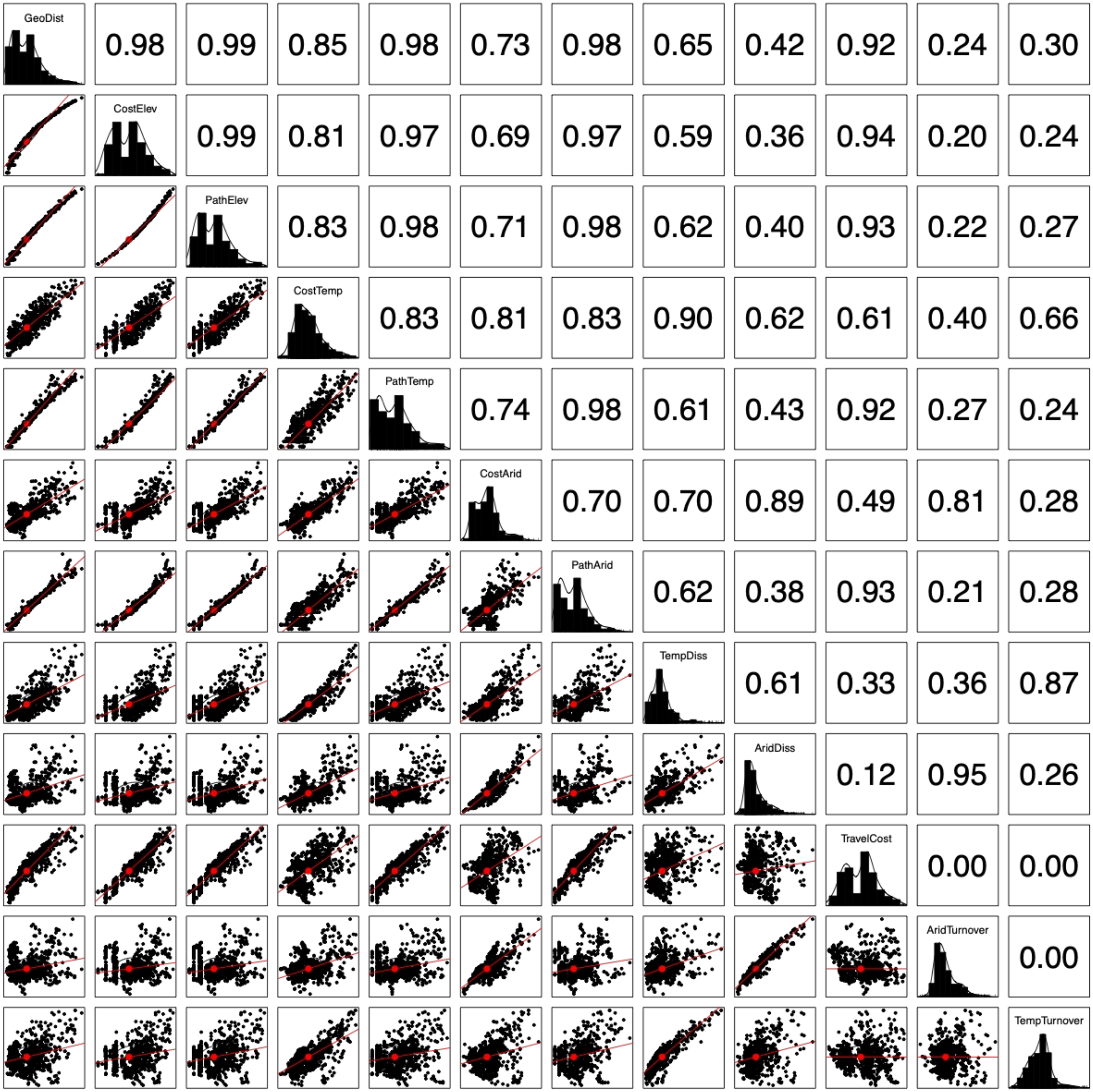

Figure S10a.
Pairs plots to show the correlation among raw variables (Figure S1b, green box) and variables reduced by PCA (Figure S1b, burgundy box). Panel a) shows variables related to the close-range analysis.

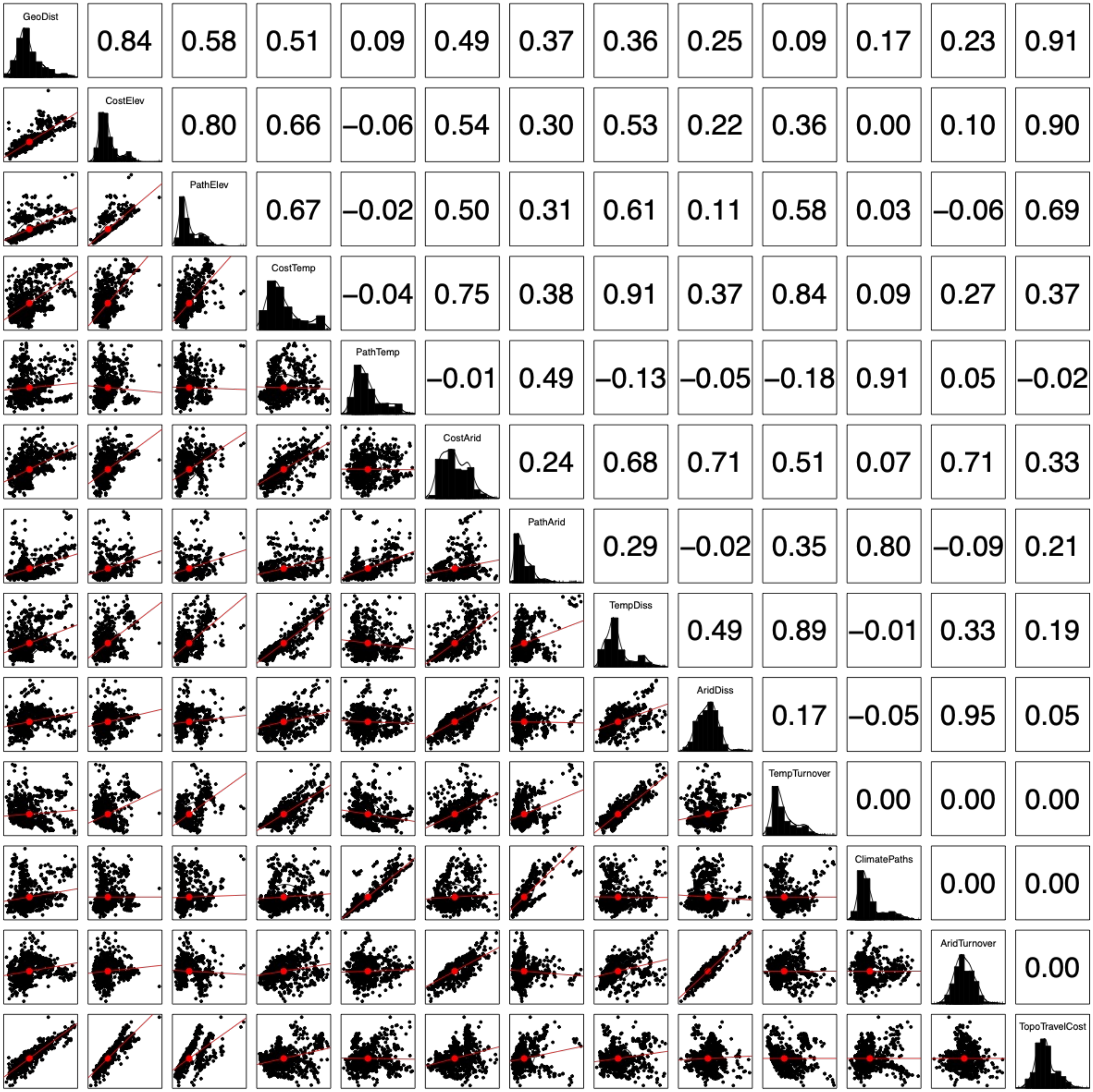

Figure S10b.
Pairs plots to show the correlation among raw variables (Figure S1b, green box) and variables reduced by PCA (Figure S1b, burgundy box). Panel b) shows variables related to the long-range analysis.

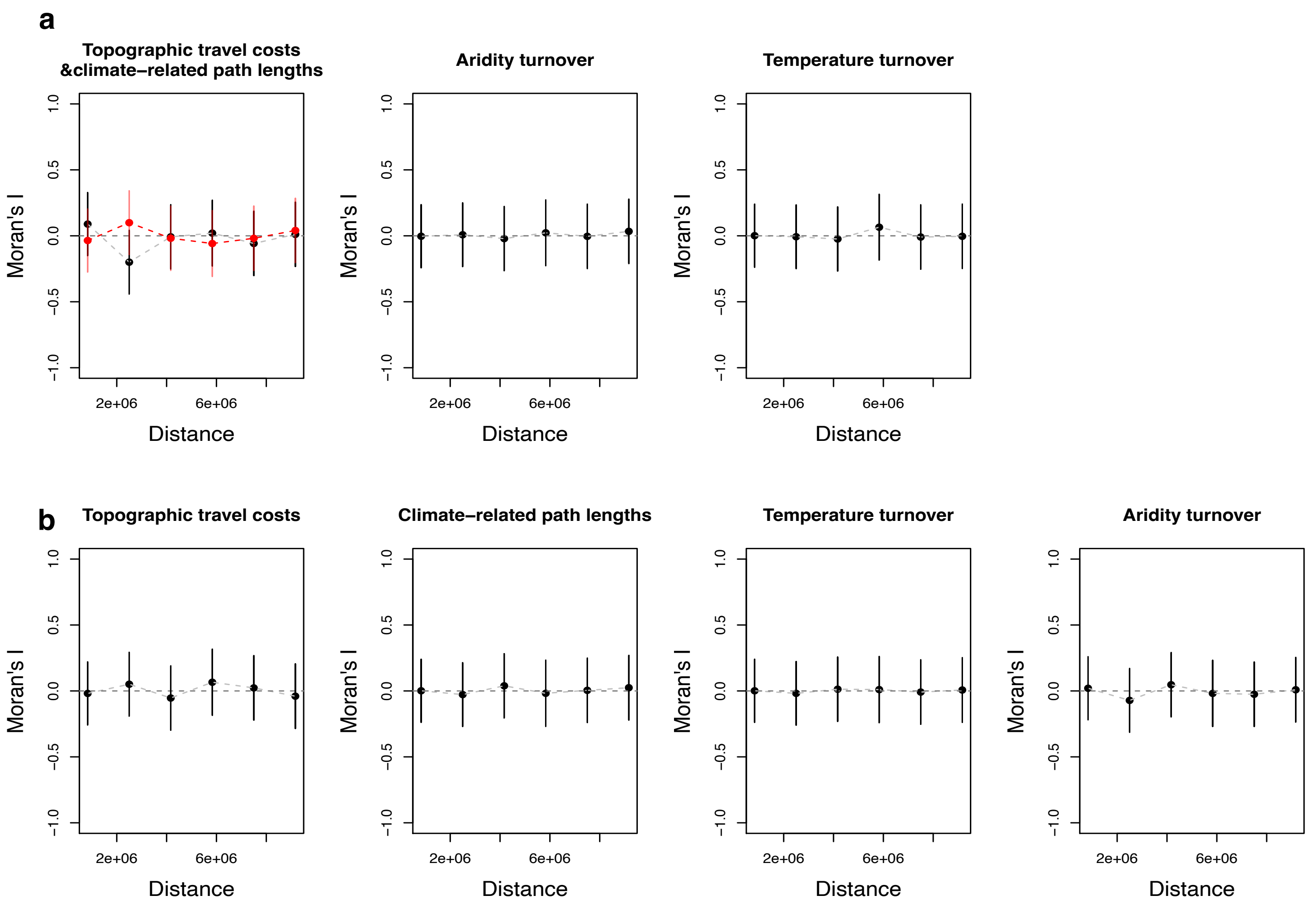

Figure S11.
Moran’s I spatial autocorrelograms of residuals corresponding to models presented in Figure 4. Each point represents a distance category, and circles close to zero indicate no/low levels of spatial autorcorrelation in model residuals. A spatial filtering approach was used if needed to correct for spatial dependence (depicted in red). Panels show results for close- (a) and long-range (b) scales.

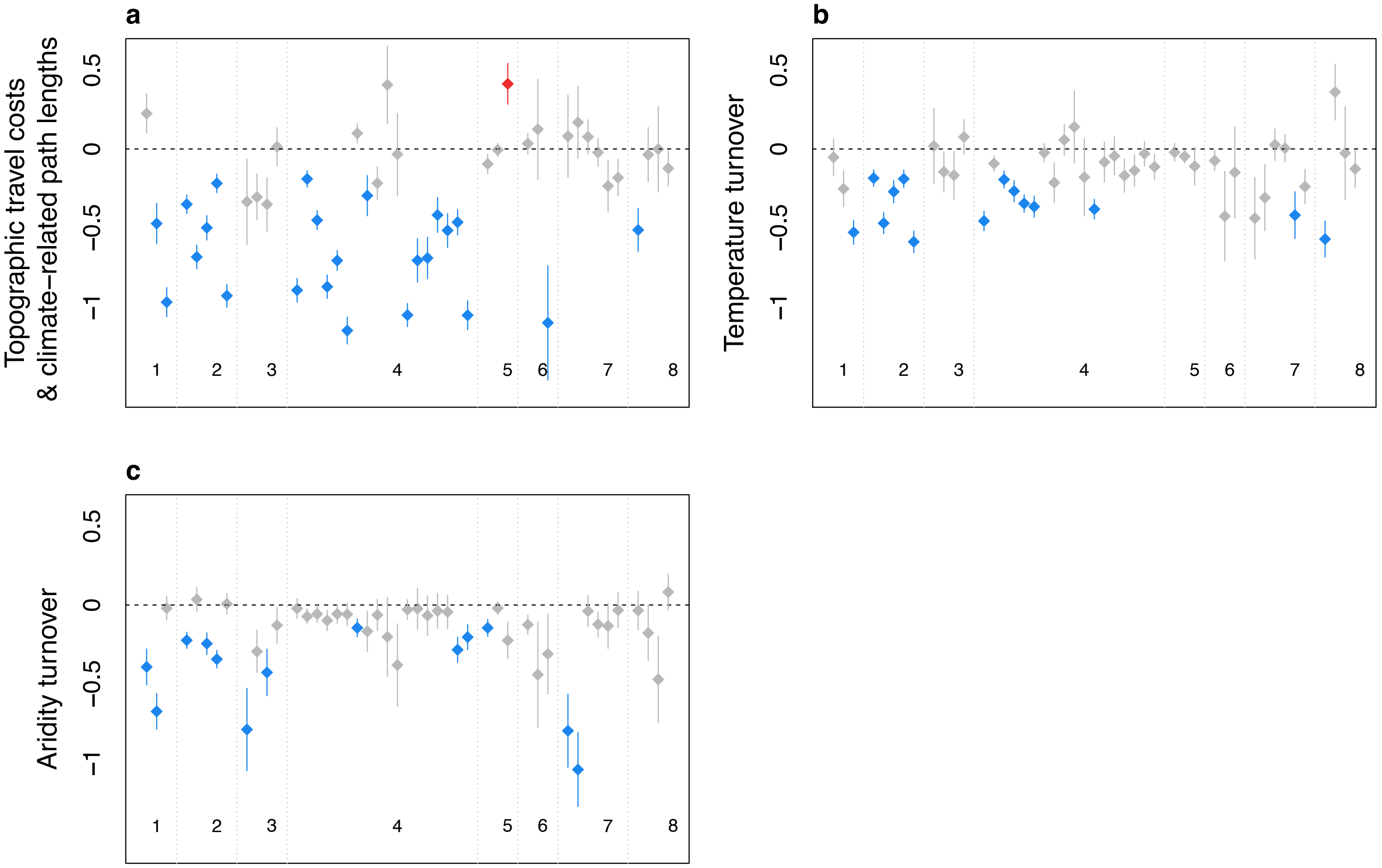

Figure S12.
Estimated effects of principal components representing environmental and travel costs to cultural similarity between pairs of societies. Each point denotes an independent model and the point's value represents the estimated effect of the principal component capturing variation in the environmental barriers listed on the y-axis label; arrows denote the size of standard errors. Models are binned into broad cultural trait categories according to their corresponding dependent variables: subsistence (1), housing ecology (2), property (3), marriage, kinship (4), community organization (5), politics, class (6), labour (7), and ritual (8). If environmental dissimilarities are associated with a lowered potential for cultural similarity, we expect to see negative values for estimated effects. Non-significant relationships are depicted with grey, whereas significant effects are shown in red (positive) and blue (negative). P-values are adjusted for false discovery rates. This figure shows results for our sensitivity analysis, in which the strength of cultural transmission is quantified by using the ten nearest neighbours, regardless of whether cultural data for these societies exist in D-PLACE (i.e., selection of which neighbours to consider is not determined by the availability of cultural data). If cultural data did not exist for at least three of these neighbours, the pair was discarded.

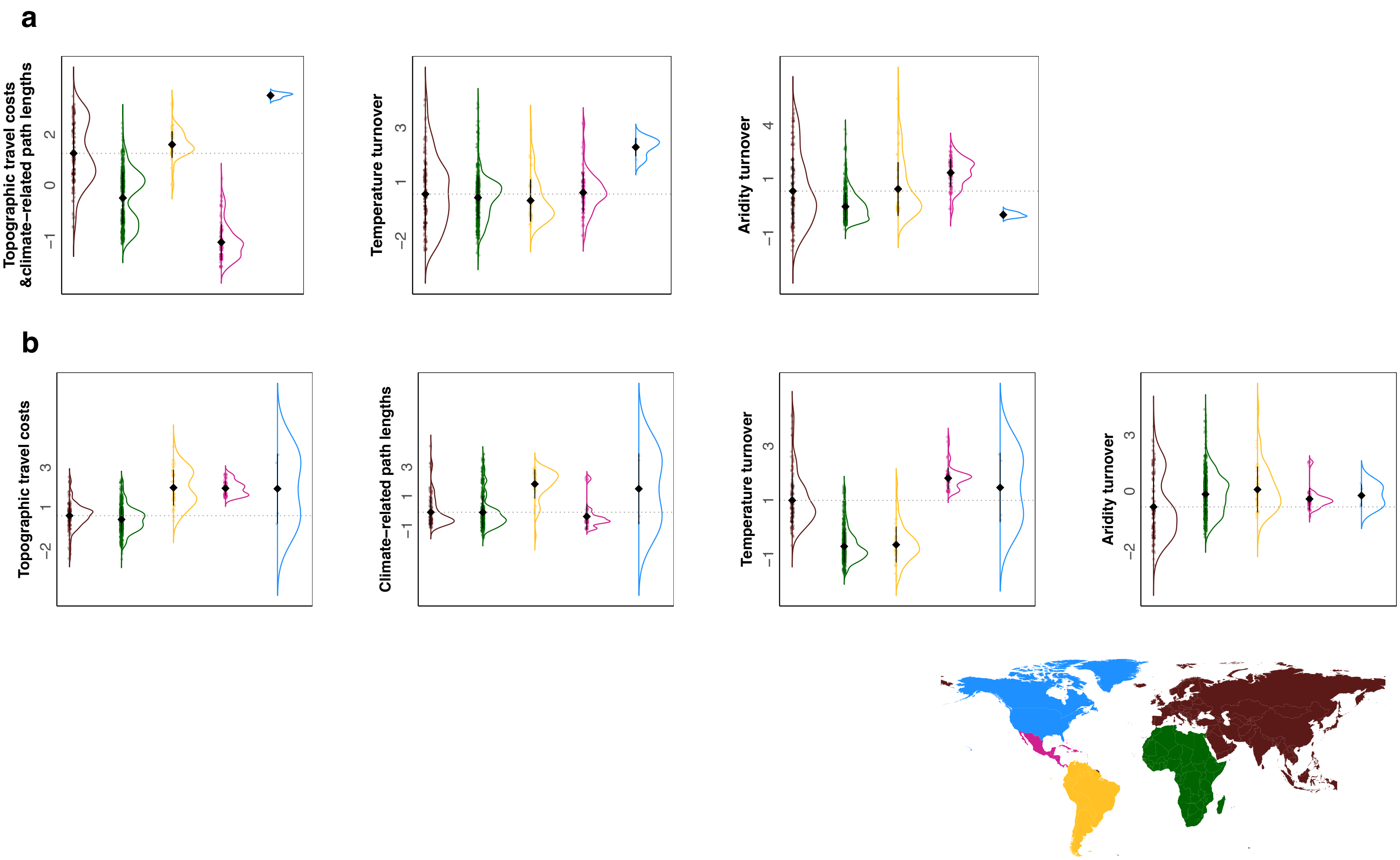

Figure S13.
The distribution of principal component scores representing candidate environmental barriers to cultural transmission. Colours highlight different major landmasses (see inset map), and panels show the distribution of environmental barriers at close- (a) and long-range (b) spatial scales.

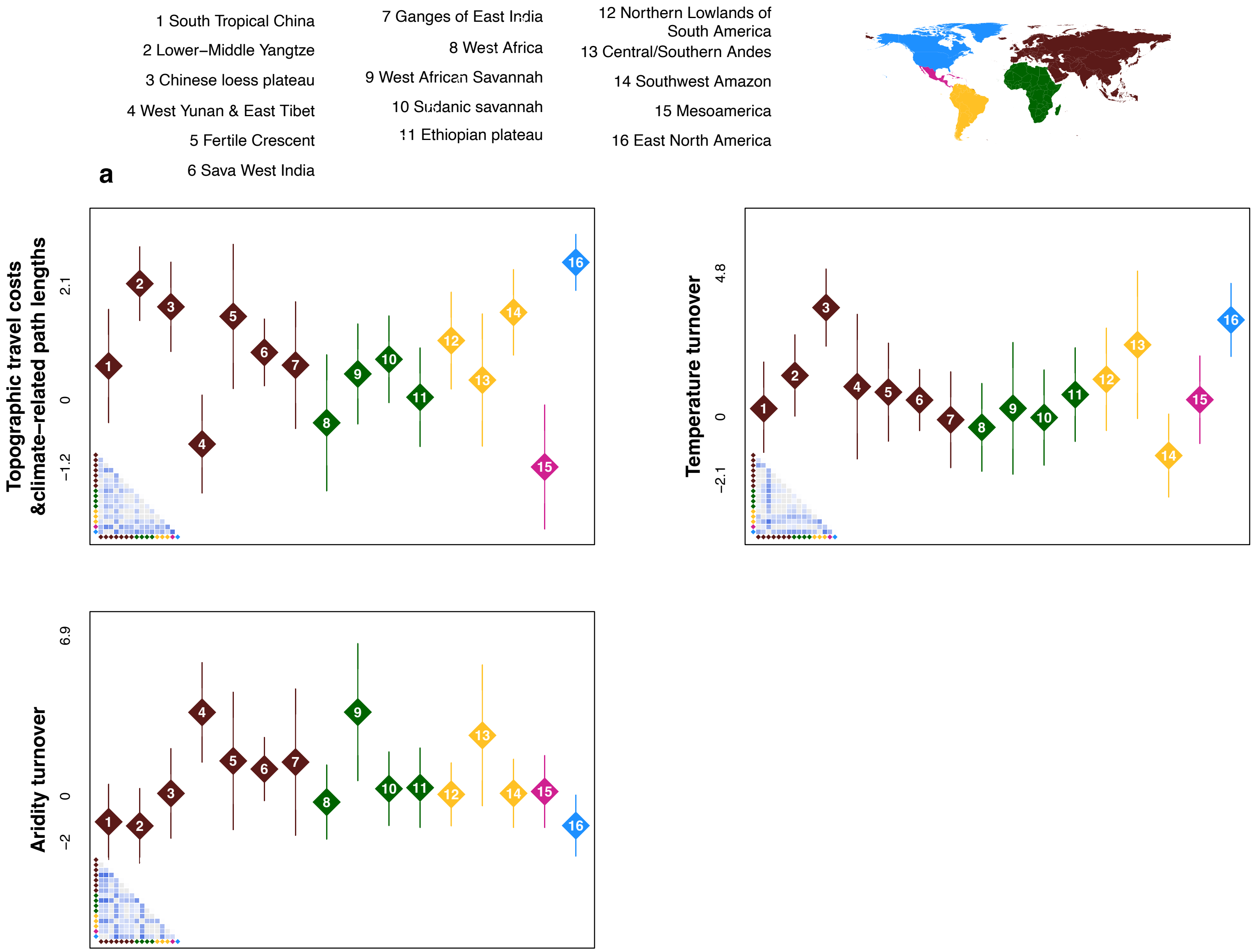

Figure S14.
Mean and standard deviation of multivariable components representing environmental costs to cultural transmission in 16 known areas of domestication origin. Each graph represents a separate model, where the dependent variable is a principal component capturing variation in the environmental barriers listed on the y-axis label. Colours highlight different major landmasses (see inset map). Non-significant differences are shown in grey. Following Diamond's hypothesis, we expect smaller means for environmental costs to cultural transmission in Eurasian areas compared to other areas of the globe. Inset matrices show pairwise Tukey's honest significant differences, with stronger blue hues depicting higher magnitudes of difference. Panels show the comparison of environmental barriers different spatial scales and using reconstructed temperature and aridity conditions from different time points.

Panel (a): Comparison of environmental barriers at close-range spatial scales. Environmental barriers are quantified using reconstructed temperature and aridity conditions from 12 kya.

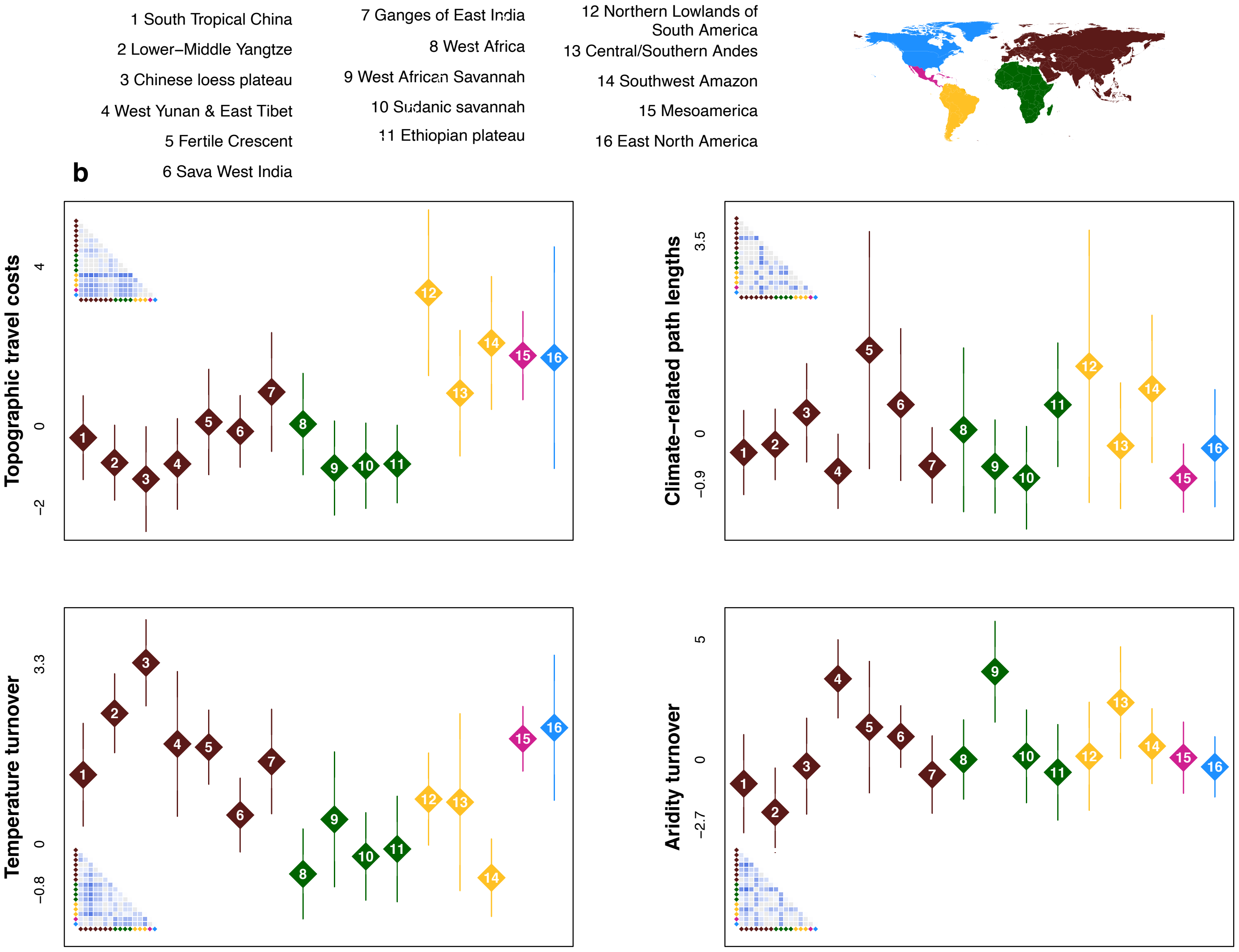

Figure S14.
Panel (b): Comparison of environmental barriers at long-range spatial scales. Environmental barriers are quantified using reconstructed temperature and aridity conditions from 12 kya.

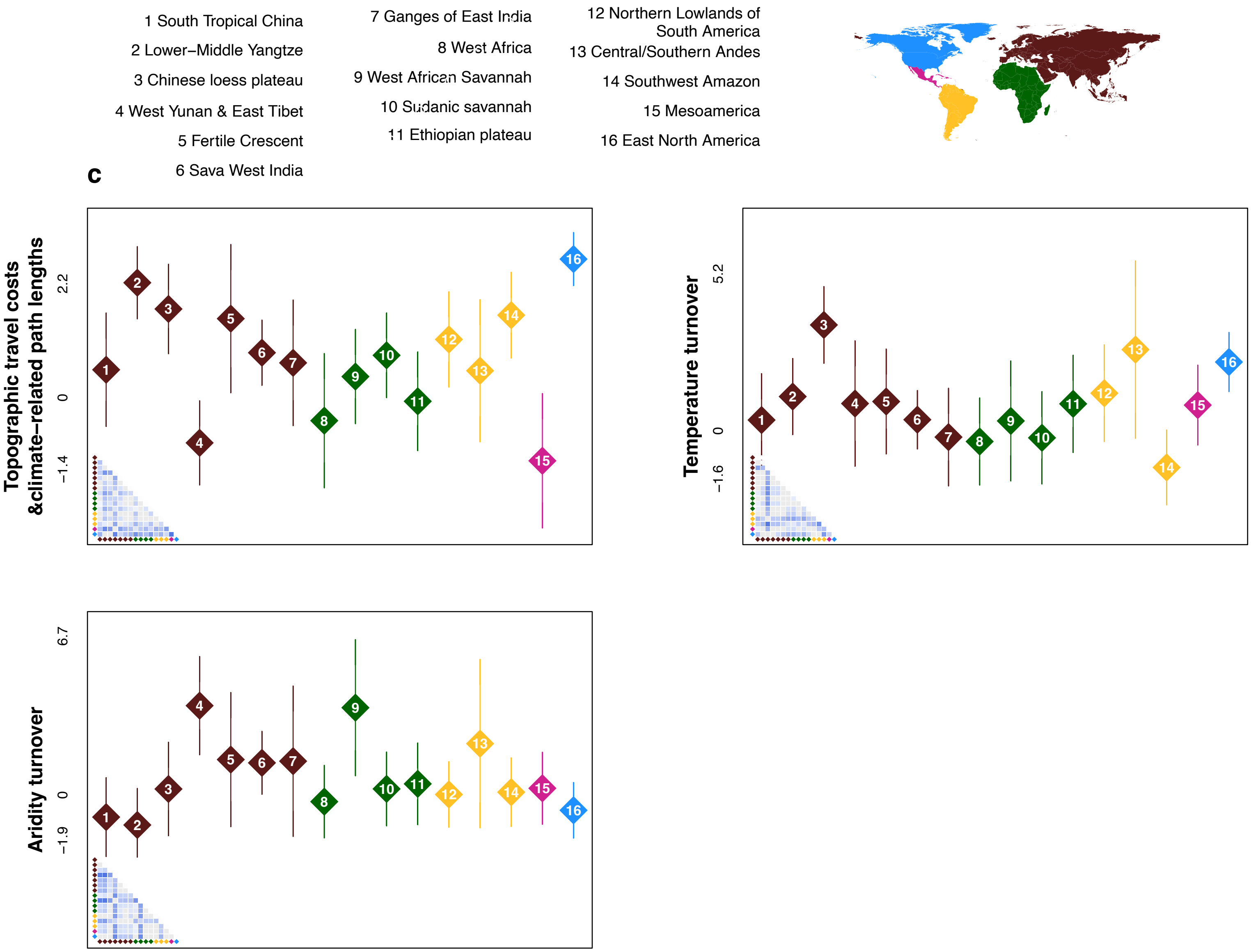

Figure S14.
Panel (c): Comparison of environmental barriers at close-range spatial scales. Environmental barriers are quantified using reconstructed temperature and aridity conditions from 8 kya.

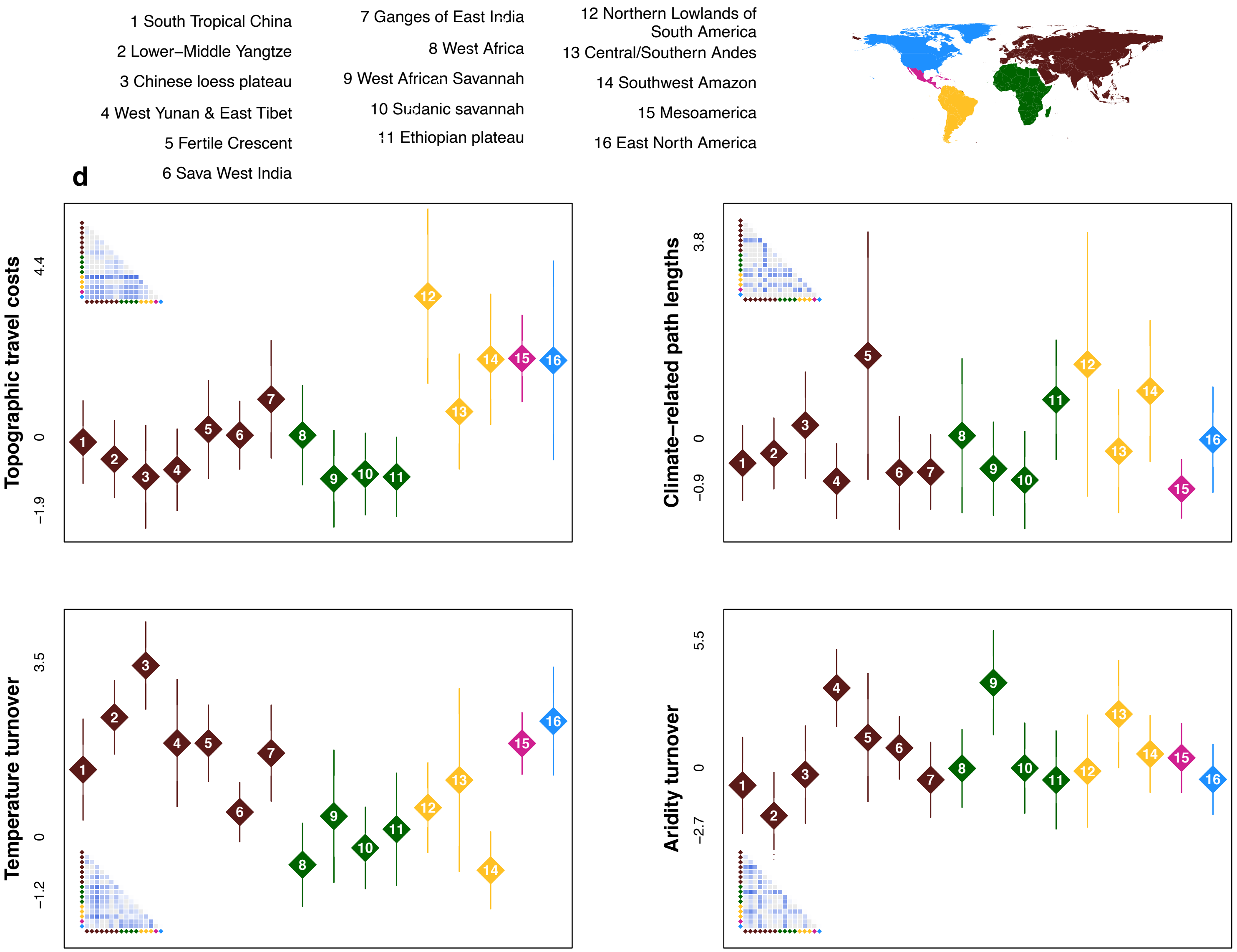

Figure S14.
Panel (d): Comparison of environmental barriers at long-range spatial scales. Environmental barriers are quantified using reconstructed temperature and aridity conditions from 8 kya.

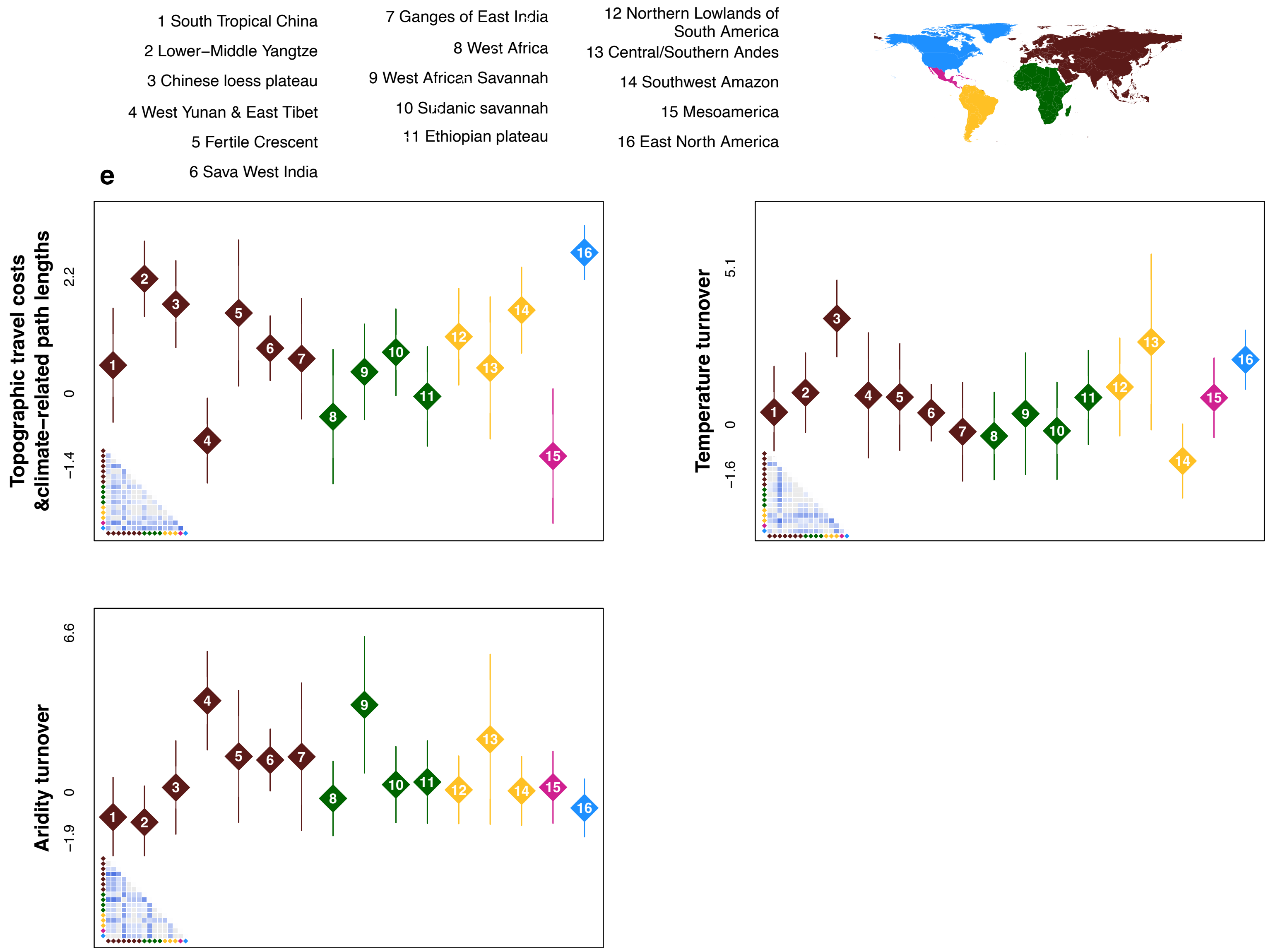

Figure S14.

Panel (e): Comparison of environmental barriers at close-range spatial scales. Environmental barriers are quantified using reconstructed temperature and aridity conditions from 4 kya.

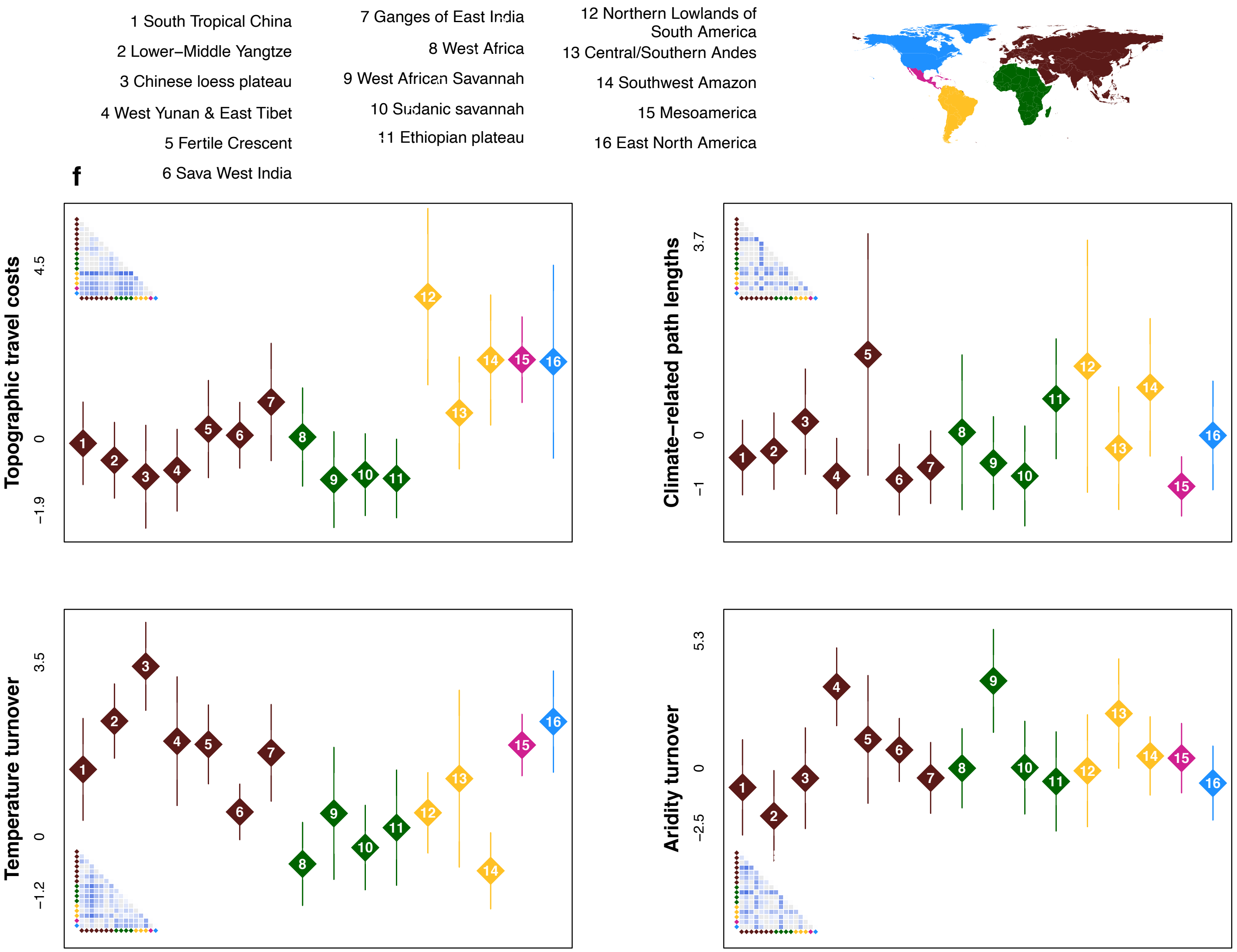

Figure S14.

Panel (f): Comparison of environmental barriers at long-range spatial scales. Environmental barriers are quantified using reconstructed temperature and aridity conditions from 4 kya.

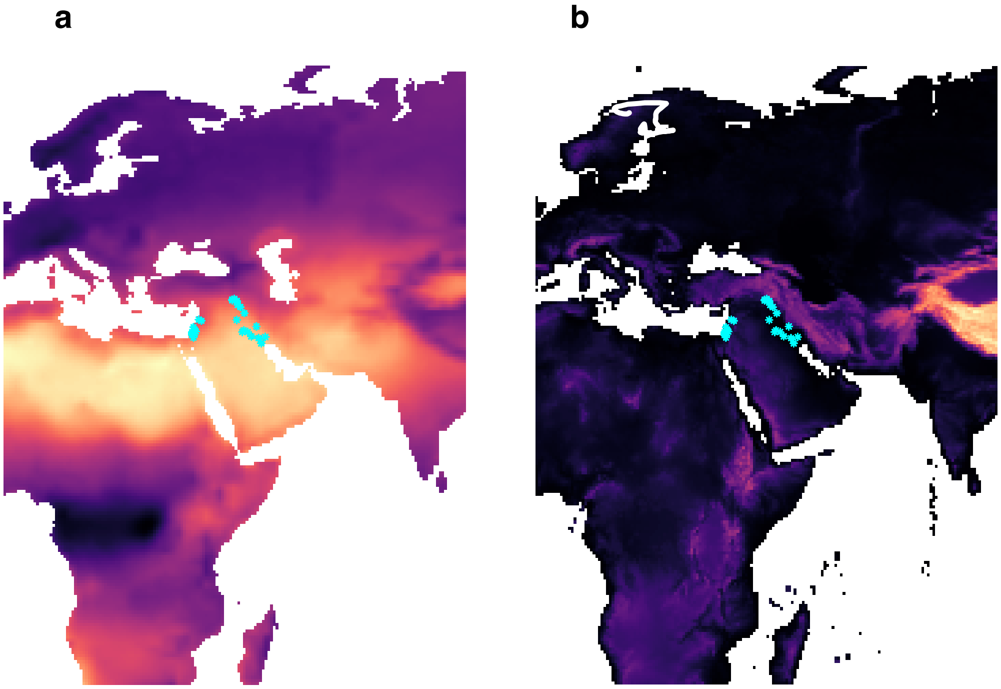

Figure S15.
Aridity index (a) and elevation (b) maps. Dark colours on the map indicate cells of low aridity index values (a), or low elevation (b). Note that societies located in the Fertile Crescent (shown in cyan) are closely gated by areas of high aridity, particularly in the south, as well as areas of high elevation in the north and north-east.

Supplementary tables

Geography is not destiny: A quantitative test of Diamond's axis of orientation hypothesis

Angela M. Chira, Russell D. Gray, Carlos A. Botero

This document includes: Tables S1, S3, S5 to S7

Other Supplementary Materials for this manuscript include the following:
Data S1 – S3 (the legends for these files are included in this document), Tables S2, S4. These files can be found at:
https://zenodo.org/records/10401227?token=eyJhbGciOiJIUzUxMiJ9.eyJpZCI6IjU3ZmFlN2RmLThiOGItNDkxZS04Y2NlLTczMzU3MDA4MDA3NSIsImRhdGEiOnt9LCJyYW5kb20iOiIxNzc2MzZlNmE5ZDMwM2VhODA5YTk3NjI0NzI3NDRlYSJ9.as8dxnbiqCSSmu7UolqGSNT9f7cSybRk6nDYtdNVQ5GwaUzdwIkj-gv8GWKpmUJExrlVnjuIeWBPTQP_jux3rg

Table S1. PCA variable loadings capturing global variation in annual mean, variation, and predictability of temperature, and mean, coefficient of variation, and predictability of precipitation. The proportion of variation captured by each PC is listed in the last row. When computing the metrics for temperature harshness and aridity index, the scores were multiplied by (-1), so that harsh temperature environments would have small means (i.e., cold temperature), high variation, and low predictability. The same was done for the aridity index.

	PC1 loadings (Temperature harshness)	PC2 loadings (Aridity index)
Temperature mean	0.88	-0.29
Temperature variation	-0.96	0.02
Temperature predictability	0.96	-0.11
Precipitation mean	0.61	0.70
Precipitation coefficient of variation	0.33	-0.86
Precipitation predictability	-0.12	0.94
Proportion variation	0.59	0.41

Table S2. (separate file due to size of table)

PCA variable loadings capturing variation in candidate barriers to cultural spread. A separate PCA is ran for each trait (indicated in column "Trait code"). The "Predictor" column indicates the raw variables that are ran in each PCA: geo-environmental barriers to cultural spread, the potential for cultural similarity in the pair due to indirect cultural transmission from other nearby neighbours (dubbed "indirect exposure"), and relatedness. The principal components are dubbed "RC" because the PCA we ran was varimax rotated, hence "R" – rotated. Different runs of the data include: (a) The strength of indirect exposure is quantified by using the five nearest neighbours with available cultural data in the D-PLACE database, and (b) The strength of indirect exposure is quantified by using the ten nearest neighbours; if cultural data did not exist for at least three of these neighbours, the pair was discarded; relatedness is quantified by using shared family ancestry.

In Table S2a – the trait EA068 is singled out. This is because, for this trait, the loadings of the components on the raw variables differ from the rest of the traits. Specifically, component 1 contains: geodesic distance, travel costs (path and accumulated cost), climate-related path lengths and aridity least-cost path cost (in all other traits, aridity least-cost path is part of another component, aridity turnover). Similar situations are encountered for Table S2b: EA028 and EA048. All these are attached as separate files.

Table S3. PCA variable loadings capturing variation in candidate barriers to cultural spread. Each data point reflects a society located in one of 17 known areas of origin of domestication, for which we compute and average barriers to cultural transmission across (a) close-range (i.e., nearest 100), and (b) long-range (i.e., nearest 100 societies located at least 2,500 km away) neighbours. The proportion of variation captured by each PC is listed in the last row. We dub the close-range PCs as: crPC1 = Topographic travel costs and climate-related path lengths, crPC2 = Aridity turnover, crPC3 = Temperature turnover, lrPC1 = Temperature turnover, lrPC2 = Climate-related path lengths, lrPC3 = Aridity turnover, lrPC4 = Topographic travel costs.

(a) Close-range scale	crPC1 loadings	crPC2 loadings	crPC3 loadings
Sqrt Geodesic distance	0.92	0.24	0.30
Log topographic travel accumulated cost	0.94	0.20	0.23
Log length topographic travel least-cost path	0.93	0.22	0.27
Log temperature-related accumulated cost	0.61	0.40	0.66
Log length temperature-related least-cost path	0.92	0.27	0.24
Log aridity-related accumulated cost	0.49	0.81	0.28
Log length aridity-related least-cost path	0.93	0.21	0.28
Sqrt Temperature dissimilarity	0.33	0.36	0.87
Sqrt Aridity dissimilarity	0.12	0.95	0.26
Proportion Variation	0.461	0.235	0.188

(b) Long-range scale	lrPC1 loadings	lrPC2 loadings	lrPC3 loadings	lrPC4 loadings
Sqrt Geodesic distance	0.09	0.17	0.23	0.91
Log topographic travel accumulated cost	0.36	0	0.1	0.9
Log length topographic travel least-cost path	0.58	0.03	-0.06	0.69
Log temperature-related accumulated cost	0.84	0.09	0.27	0.37
Log length temperature-related least-cost path	-0.18	0.91	0.05	-0.02
Log aridity-related accumulated cost	0.51	0.07	0.71	0.33
Log length aridity-related least-cost path	0.35	0.8	-0.09	0.21
Sqrt Temperature dissimilarity	0.89	-0.01	0.33	0.19
Sqrt Aridity dissimilarity	0.17	-0.05	0.95	0.05
Proportion Variation	0.269	0.167	0.185	0.271

Table S4. (separate file)

Parameters associated with models analysing predictors for cultural sharing between pairs of societies. Each row represents one model (i.e., a separate cultural trait, as indicated in the columns "Trait code" and "Trait name". The coefficients, standard errors, and p-values for each of the five predictors included in each model ('Variables reduced by PCA" in the burgundy box in Figure 1 and Figure S1a) are given. The potential for cultural similarity in the pair due to indirect cultural transmission from other nearby neighbours is dubbed "indirect exposure". Different runs of the data include: (a) The strength of indirect exposure is quantified by using the five nearest neighbours with available cultural data in the D-PLACE database, and (b) The strength of indirect exposure is quantified by using the ten nearest neighbours; if cultural data did not exist for at least three of these neighbours, the pair was discarded; relatedness is quantified by using shared family ancestry.

In Table S4a – the trait EA068 is singled out. This is because, for this trait, the loadings of the components on the raw variables differ from the rest of the traits. Specifically, component 1 contains: geodesic distance, travel costs (path and accumulated cost), climate-related path lengths and aridity least-cost path cost (in all other traits, aridity least-cost path is part of another component, aridity turnover). See Table S2a_EA068 for the loadings associated with this trait. This trait is not reported in Figure 3, due to inconsistency in labels. But the results are attached in a separate table (TableS4b_EA068.csv) Similar situations are encountered for Table S4b: EA048 and EA028. All these are attached as separate files.

Table S5. Differences in the mean strength of candidate environmental barriers to cultural spread between 16 (South India excluded) known areas of origin of domestication. Each data point reflects a society located in one of the areas of independent domestication, for which we compute and average barriers to cultural transmission across (a) close-range (i.e., nearest 100), and (b) long-range (i.e., nearest 100 societies located at least 2,500 km away) neighbours. The level of comparison is South Tropical China.

(a) Close-range scale

crPC1 – Topographic travel costs & climate-related path lengths

	Estimate	SE	p-value
Intercept	0.738	0.042	0
Lower-Middle Yangtze	1.42	0.063	0
Chinese loess plateau	1.244	0.069	0
West Yunan & East Tibet	-1.012	0.122	0
Fertile Crescent	1.317	0.062	0
Sava West India	0.24	0.05	0
Ganges of East India	-0.051	0.096	0.6
West Africa	-1.086	0.048	0
West African Savannah	-0.431	0.079	0
Sudanic savannah	-0.025	0.053	0.641
Ethiopian plateau	-0.347	0.056	0
Northern Lowlands of South America	0.475	0.077	0
Central/Southern Andes	0.484	0.151	0.001
Southwest Amazon	0.671	0.053	0
Mesoamerica	-2.06	0.054	0
East North America	1.949	0.051	0

Model parameters: adjusted R-sq = 0.93, F-statistic = 861, df = 17,1153, AIC = 1276.25

crPC2 – Aridity turnover

	Estimate	SE	p-value
Intercept	-0.703	0.093	0
Lower-Middle Yangtze	-0.077	0.161	0.63
Chinese loess plateau	1.002	0.243	0
West Yunan & East Tibet	3.751	0.267	0
Fertile Crescent	3.045	0.174	0
Sava West India	1.208	0.142	0
Ganges of East India	1.159	0.167	0
West Africa	0.24	0.095	0.012
West African Savannah	2.389	0.219	0
Sudanic savannah	0.546	0.112	0
Ethiopian plateau	0.707	0.111	0
Northern Lowlands of South America	2.048	0.125	0
Central/Southern Andes	2.923	0.337	0
Southwest Amazon	0.436	0.105	0
Mesoamerica	1.899	0.106	0
East North America	0.004	0.107	0.97

Model parameters: adjusted R-sq = 0.63, F-statistic = 136, df = 15,1155, AIC = 1956.69

crPC3 – Temperature turnover

	Estimate	SE	p-value
Intercept	-0.495	0.115	0
Lower-Middle Yangtze	0.623	0.196	0.001

Chinese loess plateau	3.345	0.208	0
West Yunan & East Tibet	1.212	0.355	0.001
Fertile Crescent	-0.411	0.267	0.123
Sava West India	1.493	0.199	0
Ganges of East India	1.372	0.146	0
West Africa	0.234	0.119	0.049
West African Savannah	3.568	0.176	0
Sudanic savannah	Jan 13	0.133	0
Ethiopian plateau	0.604	0.142	0
Northern Lowlands of South America	0.407	0.293	0.165
Central/Southern Andes	1.373	0.34	0
Southwest Amazon	-0.026	0.138	0.848
Mesoamerica	0.659	0.134	0
East North America	2.593	0.226	0
Model parameters: adjusted R-sq = 0.52, F-statistic = 85, df = 15,1155, AIC = 2706.43			

(b) Long-range scale

lrPC1 – Temperature turnover

	Estimate	SE	p-value
Intercept	0.732	0.05	0
Lower-Middle Yangtze	1.13	0.102	0
Chinese loess plateau	2.306	0.185	0
West Yunan & East Tibet	0.694	0.46	0.132
Fertile Crescent	0.349	0.117	0.003
Sava West India	-0.774	0.084	0
Ganges of East India	0.446	0.092	0
West Africa	-1.358	0.052	0
West African Savannah	0.694	0.072	0
Sudanic savannah	-0.576	0.074	0
Ethiopian plateau	-0.853	0.069	0
Northern Lowlands of South America	-0.69	0.128	0
Central/Southern Andes	-0.473	0.424	0.265
Southwest Amazon	-1.241	0.091	0
Mesoamerica	1.165	0.063	0
East North America	0.845	0.596	0.156
Model parameters: adjusted R-sq = 0.85, F-statistic = 411.1, df = 15,1050, AIC = 1087.71			

lrPC2 – Climate-related path lengths

	Estimate	SE	p-value
Intercept	-0.473	0.033	0
Lower-Middle Yangtze	0.557	0.212	0.009
Chinese loess plateau	2.204	0.288	0
West Yunan & East Tibet	-0.051	0.221	0.818
Fertile Crescent	0.814	0.181	0
Sava West India	-0.059	0.076	0.438
Ganges of East India	0.149	0.088	0.091
West Africa	0.37	0.049	0
West African Savannah	0.319	0.056	0
Sudanic savannah	-0.151	0.061	0.013
Ethiopian plateau	1.18	0.14	0
Northern Lowlands of South America	0.605	0.197	0.002
Central/Southern Andes	0.934	0.43	0.03
Southwest Amazon	2.388	0.085	0
Mesoamerica	0.163	0.087	0.062
East North America	1.798	1.048	0.086
Model parameters: adjusted R-sq = 0.48, F-statistic = 68, df = 15,1050, AIC = 2371.43			

lrPC3 – Aridity turnover

	Estimate	SE	p-value
Intercept	-1.331	0.08	0
Lower-Middle Yangtze	-0.498	0.161	0.002
Chinese loess plateau	0.321	0.164	0.051
West Yunan & East Tibet	2.968	0.954	0.002
Fertile Crescent	2.515	0.232	0
Sava West India	2.776	0.091	0
Ganges of East India	2.108	0.14	0
West Africa	1.487	0.087	0
West African Savannah	4.311	0.177	0
Sudanic savannah	1.094	0.1	0
Ethiopian plateau	0.955	0.131	0
Northern Lowlands of South America	0.796	0.359	0.027
Central/Southern Andes	3.271	0.587	0
Southwest Amazon	1.475	0.139	0
Mesoamerica	1.208	0.095	0
East North America	1.364	0.263	0

Model parameters: adjusted R-sq = 0.65, F-statistic = 132, df = 15,1050, AIC = 2403.28

lrPC4 – Topographic travel costs

	Estimate	SE	p-value
Intercept	0.004	0.046	0.925
Lower-Middle Yangtze	-0.796	0.118	0
Chinese loess plateau	-1.191	0.214	0
West Yunan & East Tibet	-0.328	0.389	0.399
Fertile Crescent	0.202	0.155	0.193
Sava West India	0.02	0.101	0.84
Ganges of East India	0.647	0.199	0.001
West Africa	-0.013	0.053	0.804
West African Savannah	-1.351	0.172	0
Sudanic savannah	-1.134	0.067	0
Ethiopian plateau	-0.987	0.063	0
Northern Lowlands of South America	2.197	0.707	0.002
Central/Southern Andes	0.462	0.2	0.021
Southwest Amazon	1.725	0.163	0
Mesoamerica	1.578	0.064	0
East North America	1.552	1.049	0.139

Model parameters: adjusted R-sq = 0.70, F-statistic = 170, df = 15,1050, AIC = 1892.42

Table S6. PCA variable loadings capturing variation in candidate barriers to cultural spread. Each data point reflects a society located in one of 17 known areas of origin of domestication, for which we compute and average barriers to cultural transmission across close-range (i.e., nearest 100), and long-range (i.e., nearest 100 societies located at least 2,500 km away) neighbours. The proportion of variation captured by each PC is listed in the last row. Data computed using reconstructed values for temperature and aridity conditions at 12kya (a, b), 8kya (c, d), 4kya (e, f). Principal components stand for: crPC1 = Topographic and climate-related path lengths, crPC2 = Aridity turnover, crPC3 = Temperature turnover.

(a) Close-range scale	crRC1 loadings	crRC2 loadings	crRC3 loadings
Sqrt Geodesic distance	0.89	0.28	0.34
Log topographic travel accumulated cost	0.92	0.28	0.24
Log length topographic travel least-cost path	0.91	0.27	0.3
Log temperature-related accumulated cost	0.52	0.39	0.74
Log length temperature-related least-cost path	0.9	0.29	0.28
Log aridity-related accumulated cost	0.52	0.76	0.35
Log length aridity-related least-cost path	0.91	0.26	0.31
Sqrt Temperature dissimilarity	0.3	0.31	0.89
Sqrt Aridity dissimilarity	0.23	0.92	0.29
Proportion Variation	0.53	0.23	0.22

(b) Long-range scale	lrRC1 loadings	lrRC2 loadings	lrRC3 loadings	lrRC4 loadings
Sqrt Geodesic distance	0.09	0.14	0.2	0.09
Log topographic travel accumulated cost	0.37	0.04	0.1	0.37
Log length topographic travel least-cost path	0.71	0.04	0	0.71
Log temperature-related accumulated cost	0.91	0.01	0.21	0.91
Log length temperature-related least-cost path	-0.12	0.9	0	-0.12
Log aridity-related accumulated cost	0.32	0.05	0.82	0.32
Log length aridity-related least-cost path	0.06	0.9	0.03	0.06
Sqrt Temperature dissimilarity	0.93	-0.11	0.21	0.93
Sqrt Aridity dissimilarity	0.1	0	0.96	0.1
Proportion Variation	0.31	0.2	0.8	0.58

(c) Close-range scale	crRC1 loadings	crRC2 loadings	crRC3 loadings
Sqrt Geodesic distance	0.89	0.29	0.34
Log topographic travel accumulated cost	0.92	0.28	0.25
Log length topographic travel least-cost path	0.91	0.28	0.3
Log temperature-related accumulated cost	0.52	0.37	0.75

Log length temperature-related least-cost path	0.9	0.3	0.28	
Log aridity-related accumulated cost	0.54	0.75	0.33	
Log length aridity-related least-cost path	0.9	0.26	0.32	
Sqrt Temperature dissimilarity	0.29	0.28	0.91	
Sqrt Aridity dissimilarity	0.25	0.92	0.28	
Proportion Variation	0.53	0.23	0.22	

(d) Long-range scale	lrRC1 loadings	lrRC2 loadings	lrRC3 loadings	lrRC4 loadings
Sqrt Geodesic distance	0.03	0.15	0.23	0.91
Log topographic travel accumulated cost	0.29	0.07	0.13	0.92
Log length topographic travel least-cost path	0.67	0.02	0.05	0.62
Log temperature-related accumulated cost	0.93	0.02	0.2	0.18
Log length temperature-related least-cost path	-0.1	0.89	-0.04	0.01
Log aridity-related accumulated cost	0.3	0.05	0.82	0.38
Log length aridity-related least-cost path	0.03	0.87	0.04	0.16
Sqrt Temperature dissimilarity	0.94	-0.13	0.19	0.08
Sqrt Aridity dissimilarity	0.13	-0.03	0.95	0.06
Proportion Variation	0.3	0.2	0.22	0.28

(e) Close-range scale	crRC1 loadings	crRC2 loadings	crRC3 loadings	
Sqrt Geodesic distance	0.89	0.29	0.34	
Log topographic travel accumulated cost	0.92	0.28	0.25	
Log length topographic travel least-cost path	0.91	0.28	0.3	
Log temperature-related accumulated cost	0.53	0.36	0.75	
Log length temperature-related least-cost path	0.9	0.3	0.28	
Log aridity-related accumulated cost	0.54	0.76	0.32	
Log length aridity-related least-cost path	0.9	0.26	0.32	
Sqrt Temperature dissimilarity	0.29	0.27	0.91	
Sqrt Aridity dissimilarity	0.24	0.92	0.27	
Proportion Variation	0.53	0.23	0.22	

(f) Long-range scale	lrRC1 loadings	lrRC2 loadings	lrRC3 loadings	lrRC4 loadings
Sqrt Geodesic distance	0.03	0.16	0.23	0.9
Log topographic travel accumulated cost	0.29	0.07	0.12	0.92
Log length topographic travel least-cost path	0.66	0.02	0.03	0.62
Log temperature-related accumulated cost	0.93	0.03	0.2	0.18
Log length temperature-related least-cost path	-0.07	0.89	-0.03	0
Log aridity-related accumulated cost	0.29	0.03	0.81	0.4

Log length aridity-related least-cost path	0.01	0.87	0.04	0.18
Sqrt Temperature dissimilarity	0.94	-0.12	0.19	0.08
Sqrt Aridity dissimilarity	0.13	-0.02	0.96	0.05
Proportion Variation	0.26	0.89	0.71	0.52

Table S7. Differences in the mean strength of candidate environmental barriers to cultural spread between 16 (South India excluded) known areas of origin of domestication. Each data point reflects a society located in one of the areas of independent domestication, for which we compute and average barriers to cultural transmission across close-range (i.e., nearest 100), and long-range (i.e., nearest 100 societies located at least 2,500 km away) neighbours. The level of comparison is South Tropical China. Data computed using reconstructed values for temperature and aridity conditions at 12kya (a, b), 8kya (c, d), 4kya (e, f).

(a) Close-range scale

crPC1

	Estimate	SE	p-value
Intercept	0.626	0.055	0
Lower-Middle Yangtze	1.523	0.079	0
Chinese loess plateau	1.094	0.092	0
West Yunan & East Tibet	-1.443	0.156	0
Fertile Crescent	0.917	0.18	0
Sava West India	0.252	0.072	0.001
Ganges of East India	0.016	0.14	0.909
West Africa	-1.05	0.062	0
West African Savannah	-0.146	0.118	0.214
Sudanic savannah	0.124	0.062	0.047
Ethiopian plateau	-0.578	0.067	0
Northern Lowlands of South America	0.47	0.166	0.005
Central/Southern Andes	-0.26	0.225	0.249
Southwest Amazon	0.992	0.069	0
Mesoamerica	-1.867	0.096	0
East North America	1.916	0.059	0

Model parameters: adjusted R-sq = 0.92, F-statistic = 661, df = 17,1006, AIC = 1500.15

crPC2

	Estimate	SE	p-value
Intercept	-1.09	0.062	0
Lower-Middle Yangtze	-0.174	0.176	0.322
Chinese loess plateau	1.216	0.218	0
West Yunan & East Tibet	4.684	0.433	0
Fertile Crescent	2.606	0.456	0
Sava West India	2.259	0.12	0
Ganges of East India	2.555	0.463	0
West Africa	0.845	0.065	0
West African Savannah	4.694	0.518	0
Sudanic savannah	1.416	0.08	0
Ethiopian plateau	1.462	0.089	0
Northern Lowlands of South America	1.175	0.135	0
Central/Southern Andes	3.701	0.687	0
Southwest Amazon	Jan 22	0.086	0
Mesoamerica	1.296	0.09	0
East North America	-0.16	0.128	0.212

Model parameters: adjusted R-sq = 0.47, F-statistic = 62, df = 15,1008, AIC = 1698.37

crPC3

	Estimate	SE	p-value
Intercept	0.271	0.076	0
Lower-Middle Yangtze	1.102	0.167	0
Chinese loess plateau	3.358	0.127	0
West Yunan & East Tibet	0.731	0.592	0.217
Fertile Crescent	0.548	0.192	0.004
Sava West India	0.285	0.099	0.004
Ganges of East India	-0.37	0.173	0.033
West Africa	-0.623	0.08	0
West African Savannah	0.008	0.353	0.981
Sudanic savannah	-0.296	0.104	0.005
Ethiopian plateau	0.466	0.102	0
Northern Lowlands of South America	0.974	0.363	0.007
Central/Southern Andes	2.121	0.529	0
Southwest Amazon	-1.56	0.109	0
Mesoamerica	0.296	0.111	0.008
East North America	2.948	0.188	0
Model parameters: adjusted R-sq = 0.72, F-statistic = 158, df = 17,1006 , AIC =1879.06			

(b) Long-range scale

lrPC1

	Estimate	SE	p-value
Intercept	1.259	0.055	0
Lower-Middle Yangtze	1.118	0.098	0
Chinese loess plateau	2.037	0.098	0
West Yunan & East Tibet	0.561	0.353	0.113
Fertile Crescent	0.501	0.076	0
Sava West India	-0.734	0.096	0
Ganges of East India	0.24	0.123	0.05
West Africa	-1.805	0.057	0
West African Savannah	-0.812	0.22	0
Sudanic savannah	-1.487	0.065	0
Ethiopian plateau	-1.351	0.075	0
Northern Lowlands of South America	-0.443	0.177	0.013
Central/Southern Andes	-0.499	0.41	0.224
Southwest Amazon	-1.873	0.067	0
Mesoamerica	0.654	0.058	0
East North America	0.856	0.432	0.048
Model parameters: adjusted R-sq = 0.94, F-statistic = 1012, df = 15,1008, AIC = 804.91			

lrPC2

	Estimate	SE	p-value
Intercept	-0.344	0.036	0
Lower-Middle Yangtze	0.149	0.068	0.029
Chinese loess plateau	0.731	0.115	0
West Yunan & East Tibet	-0.346	0.095	0
Fertile Crescent	1.886	0.417	0
Sava West India	0.883	0.354	0.013
Ganges of East India	-0.235	0.062	0
West Africa	0.419	0.057	0
West African Savannah	-0.257	0.114	0.024
Sudanic savannah	-0.465	0.061	0
Ethiopian plateau	0.879	0.078	0

Northern Lowlands of South America	1.587	0.913	0.082
Central/Southern Andes	0.125	0.247	0.612
Southwest Amazon	1.171	0.144	0
Mesoamerica	-0.471	0.043	0
East North America	0.08	0.308	0.794
Model parameters: adjusted R-sq = 0.46, F-statistic = 58, df = 15,1008, AIC = 2244.31			

lrPC3			
	Estimate	SE	p-value
Intercept	-0.987	0.114	0
Lower-Middle Yangtze	-1.179	0.271	0
Chinese loess plateau	0.722	0.26	0.006
West Yunan & East Tibet	4.325	0.257	0
Fertile Crescent	2.333	0.419	0
Sava West India	1.943	0.143	0
Ganges of East India	0.375	0.168	0.026
West Africa	0.997	0.117	0
West African Savannah	4.62	0.308	0
Sudanic savannah	1.129	0.145	0
Ethiopian plateau	0.468	0.148	0.002
Northern Lowlands of South America	1.131	0.54	0.037
Central/Southern Andes	3.345	0.458	0
Southwest Amazon	1.549	0.138	0
Mesoamerica	1.073	0.129	0
East North America	0.694	0.15	0
Model parameters: adjusted R-sq = 0.43, F-statistic = 53, df = 15,1008, AIC = 2092.73			

lrPC4			
	Estimate	SE	p-value
Intercept	-0.284	0.048	0
Lower-Middle Yangtze	-0.62	0.107	0
Chinese loess plateau	-1.026	0.174	0
West Yunan & East Tibet	-0.652	0.207	0.002
Fertile Crescent	0.388	0.171	0.023
Sava West India	0.156	0.109	0.155
Ganges of East India	1.131	0.194	0
West Africa	0.337	0.055	0
West African Savannah	-0.752	0.156	0
Sudanic savannah	-0.696	0.065	0
Ethiopian plateau	-0.653	0.059	0
Northern Lowlands of South America	3.591	0.639	0
Central/Southern Andes	1.102	0.331	0.001
Southwest Amazon	2.346	0.166	0
Mesoamerica	2.034	0.081	0
East North America	1.982	1.059	0.062
Model parameters: adjusted R-sq = 0.68, F-statistic = 144, df = 15,1008, AIC = 1732.08			

(c) Close-range scale			
crPC1			
	Estimate	SE	p-value
Intercept	0.534	0.057	0

	Estimate	SE	p-value
Lower-Middle Yangtze	1.659	0.078	0
Chinese loess plateau	1.158	0.095	0
West Yunan & East Tibet	-1.393	0.122	0
Fertile Crescent	0.974	0.199	0
Sava West India	0.325	0.072	0
Ganges of East India	0.132	0.143	0.357
West Africa	-0.97	0.064	0
West African Savannah	-0.13	0.111	0.242
Sudanic savannah	0.276	0.064	0
Ethiopian plateau	-0.601	0.069	0
Northern Lowlands of South America	0.578	0.167	0.001
Central/Southern Andes	-0.018	0.245	0.94
Southwest Amazon	1.04	0.072	0
Mesoamerica	-1.735	0.128	0
East North America	2.109	0.058	0
Model parameters: adjusted R-sq = 0.95, F-statistic = 1186, df = 17,985, AIC = 1497.07			

crPC2

	Estimate	SE	p-value
Intercept	-0.924	0.07	0
Lower-Middle Yangtze	-0.331	0.165	0.045
Chinese loess plateau	1.18	0.236	0
West Yunan & East Tibet	4.676	0.415	0
Fertile Crescent	2.413	0.444	0
Sava West India	2.276	0.121	0
Ganges of East India	2.346	0.474	0
West Africa	0.649	0.072	0
West African Savannah	4.591	0.504	0
Sudanic savannah	1.183	0.086	0
Ethiopian plateau	1.395	0.097	0
Northern Lowlands of South America	0.95	0.158	0
Central/Southern Andes	3.082	0.828	0
Southwest Amazon	1.046	0.092	0
Mesoamerica	1.21	0.105	0
East North America	0.286	0.088	0.001
Model parameters: adjusted R-sq = 0.47, F-statistic = 61, df = 15,987, AIC = 1593.32			

crPC3

	Estimate	SE	p-value
Intercept	0.355	0.081	0
Lower-Middle Yangtze	0.772	0.157	0
Chinese loess plateau	3.127	0.135	0
West Yunan & East Tibet	0.543	0.51	0.287
Fertile Crescent	0.609	0.238	0.011
Sava West India	0.011	0.095	0.91
Ganges of East India	-0.561	0.192	0.004
West Africa	-0.706	0.085	0
West African Savannah	-0.03	0.327	0.928
Sudanic savannah	-0.585	0.109	0
Ethiopian plateau	0.532	0.113	0
Northern Lowlands of South America	0.882	0.35	0.012
Central/Southern Andes	2.315	0.7	0.001

Southwest Amazon	-1.559	0.102	0
Mesoamerica	0.491	0.117	0
East North America	1.908	0.108	0
Model parameters: adjusted R-sq = 0.75, F-statistic = 199, df = 15,987, AIC = 1911.5			

(d) Long-range scale			
lrPC1			
	Estimate	SE	p-value
Intercept	1.333	0.059	0
Lower-Middle Yangtze	1.034	0.092	0
Chinese loess plateau	2.059	0.108	0
West Yunan & East Tibet	0.524	0.314	0.096
Fertile Crescent	0.522	0.086	0
Sava West India	-0.841	0.067	0
Ganges of East India	0.325	0.117	0.005
West Africa	-1.883	0.06	0
West African Savannah	-0.922	0.231	0
Sudanic savannah	-1.55	0.067	0
Ethiopian plateau	-1.181	0.086	0
Northern Lowlands of South America	-0.754	0.183	0
Central/Southern Andes	-0.208	0.44	0.637
Southwest Amazon	-1.993	0.071	0
Mesoamerica	0.518	0.062	0
East North America	0.959	0.288	0.001
Model parameters: adjusted R-sq = 0.94, F-statistic = 977, df = 15,988, AIC = 720.31			

lrPC2			
	Estimate	SE	p-value
Intercept	-0.473	0.027	0
Lower-Middle Yangtze	0.187	0.067	0.006
Chinese loess plateau	0.729	0.136	0
West Yunan & East Tibet	-0.346	0.097	0
Fertile Crescent	2.065	0.473	0
Sava West India	-0.184	0.228	0.419
Ganges of East India	-0.171	0.058	0.003
West Africa	0.527	0.05	0
West African Savannah	-0.107	0.116	0.359
Sudanic savannah	-0.324	0.054	0
Ethiopian plateau	1.219	0.073	0
Northern Lowlands of South America	1.898	0.914	0.038
Central/Southern Andes	0.229	0.233	0.327
Southwest Amazon	1.385	0.138	0
Mesoamerica	-0.495	0.03	0
East North America	0.451	0.264	0.088
Model parameters: adjusted R-sq = 0.61, F-statistic = 104, df = 15,988, AIC = 2096.31			

lrPC3			
	Estimate	SE	p-value
Intercept	-0.744	0.113	0
Lower-Middle Yangtze	-1.301	0.27	0
Chinese loess plateau	0.462	0.275	0.093
West Yunan & East Tibet	4.168	0.255	0
Fertile Crescent	2.044	0.43	0

Sava West India	1.603	0.146	0
Ganges of East India	0.234	0.162	0.151
West Africa	0.723	0.116	0
West African Savannah	4.392	0.335	0
Sudanic savannah	0.736	0.143	0
Ethiopian plateau	0.229	0.154	0.137
Northern Lowlands of South America	0.61	0.598	0.308
Central/Southern Andes	3.051	0.42	0
Southwest Amazon	1.344	0.143	0
Mesoamerica	1.177	0.132	0
East North America	0.255	0.239	0.287
Model parameters: adjusted R-sq = 0.41, F-statistic = 48, df = 15,988, AIC = 2083.39			

lrPC4			
	Estimate	SE	p-value
Intercept	-0.124	0.049	0.011
Lower-Middle Yangtze	-0.442	0.117	0
Chinese loess plateau	-0.898	0.176	0
West Yunan & East Tibet	-0.717	0.171	0
Fertile Crescent	0.33	0.16	0.04
Sava West India	0.176	0.098	0.073
Ganges of East India	1.108	0.2	0
West Africa	0.176	0.055	0.002
West African Savannah	-0.949	0.172	0
Sudanic savannah	-0.827	0.064	0
Ethiopian plateau	-0.902	0.062	0
Northern Lowlands of South America	3.774	0.717	0
Central/Southern Andes	0.789	0.282	0.005
Southwest Amazon	2.14	0.168	0
Mesoamerica	2.164	0.094	0
East North America	2.111	0.957	0.028
Model parameters: adjusted R-sq = 0.67, F-statistic = 135, df = 15,988, AIC = 1706.72			

(e) Close-range scale			
crPC1			
	Estimate	SE	p-value
Intercept	0.626	0.055	0
Lower-Middle Yangtze	1.523	0.079	0
Chinese loess plateau	1.094	0.092	0
West Yunan & East Tibet	-1.443	0.156	0
Fertile Crescent	0.917	0.18	0
Sava West India	0.252	0.072	0.001
Ganges of East India	0.016	0.14	0.909
West Africa	-1.05	0.062	0
West African Savannah	-0.146	0.118	0.214
Sudanic savannah	0.124	0.062	0.047
Ethiopian plateau	-0.578	0.067	0
Northern Lowlands of South America	0.47	0.166	0.005
Central/Southern Andes	-0.26	0.225	0.249
Southwest Amazon	0.992	0.069	0
Mesoamerica	-1.867	0.096	0
East North America	1.916	0.059	0

Model parameters: adjusted R-sq = 0.92, F-statistic = 661, df = 17,1006, AIC = 1500.15			

crPC2			
	Estimate	SE	p-value
Intercept	-1.09	0.062	0
Lower-Middle Yangtze	-0.174	0.176	0.322
Chinese loess plateau	1.216	0.218	0
West Yunan & East Tibet	4.684	0.433	0
Fertile Crescent	2.606	0.456	0
Sava West India	2.259	0.12	0
Ganges of East India	2.555	0.463	0
West Africa	0.845	0.065	0
West African Savannah	4.694	0.518	0
Sudanic savannah	1.416	0.08	0
Ethiopian plateau	1.462	0.089	0
Northern Lowlands of South America	1.175	0.135	0
Central/Southern Andes	3.701	0.687	0
Southwest Amazon	Jan 22	0.086	0
Mesoamerica	1.296	0.09	0
East North America	-0.16	0.128	0.212
Model parameters: adjusted R-sq = 0.47, F-statistic = 62, df = 15,1008, AIC = 1698.37			

crPC3			
	Estimate	SE	p-value
Intercept	0.271	0.076	0
Lower-Middle Yangtze	1.102	0.167	0
Chinese loess plateau	3.358	0.127	0
West Yunan & East Tibet	0.731	0.592	0.217
Fertile Crescent	0.548	0.192	0.004
Sava West India	0.285	0.099	0.004
Ganges of East India	-0.37	0.173	0.033
West Africa	-0.623	0.08	0
West African Savannah	0.008	0.353	0.981
Sudanic savannah	-0.296	0.104	0.005
Ethiopian plateau	0.466	0.102	0
Northern Lowlands of South America	0.974	0.363	0.007
Central/Southern Andes	2.121	0.529	0
Southwest Amazon	-1.56	0.109	0
Mesoamerica	0.296	0.111	0.008
East North America	2.948	0.188	0
Model parameters: adjusted R-sq = 0.72, F-statistic = 158, df = 17,1006 , AIC = 1879.06			

(f) Long-range scale			
lrPC1			
	Estimate	SE	p-value
Intercept	1.337	0.06	0
Lower-Middle Yangtze	0.964	0.095	0
Chinese loess plateau	2.056	0.11	0
West Yunan & East Tibet	0.563	0.323	0.081
Fertile Crescent	0.499	0.092	0
Sava West India	-0.848	0.062	0
Ganges of East India	0.331	0.118	0.005
West Africa	-1.885	0.061	0

	Estimate	SE	p-value
West African Savannah	-0.877	0.232	0
Sudanic savannah	-1.559	0.069	0
Ethiopian plateau	-1.162	0.089	0
Northern Lowlands of South America	-0.865	0.145	0
Central/Southern Andes	-0.184	0.423	0.664
Southwest Amazon	-2.022	0.071	0
Mesoamerica	0.486	0.062	0
East North America	0.953	0.258	0
Model parameters: adjusted R-sq = 0.94, F-statistic = 971, df = 15,992, AIC = 748.52			

lrPC2			
	Estimate	SE	p-value
Intercept	-0.432	0.027	0
Lower-Middle Yangtze	0.125	0.083	0.135
Chinese loess plateau	0.696	0.135	0
West Yunan & East Tibet	-0.359	0.102	0
Fertile Crescent	1.996	0.462	0
Sava West India	-0.428	0.078	0
Ganges of East India	-0.186	0.054	0.001
West Africa	0.489	0.05	0
West African Savannah	-0.107	0.116	0.359
Sudanic savannah	-0.356	0.056	0
Ethiopian plateau	1.138	0.073	0
Northern Lowlands of South America	1.768	0.872	0.043
Central/Southern Andes	0.181	0.234	0.438
Southwest Amazon	1.359	0.134	0
Mesoamerica	-0.557	0.031	0
East North America	0.428	0.282	0.129
Model parameters: adjusted R-sq = 0.6, F-statistic = 102, df = 15,992, AIC = 2120.48			

lrPC3			
	Estimate	SE	p-value
Intercept	-0.802	0.109	0
Lower-Middle Yangtze	-1.193	0.286	0
Chinese loess plateau	0.386	0.283	0.173
West Yunan & East Tibet	4.208	0.255	0
Fertile Crescent	2.008	0.417	0
Sava West India	1.566	0.146	0
Ganges of East India	0.398	0.144	0.006
West Africa	0.801	0.112	0
West African Savannah	4.456	0.322	0
Sudanic savannah	0.829	0.142	0
Ethiopian plateau	0.25	0.15	0.096
Northern Lowlands of South America	0.696	0.583	0.232
Central/Southern Andes	3.088	0.423	0
Southwest Amazon	1.316	0.141	0
Mesoamerica	1.224	0.129	0
East North America	0.18	0.266	0.498
Model parameters: adjusted R-sq = 0.4, F-statistic = 46, df = 15,992, AIC = 2107.11			

lrPC4			
	Estimate	SE	p-value

Intercept	-0.119	0.048	0.014
Lower-Middle Yangtze	-0.44	0.114	0
Chinese loess plateau	-0.868	0.176	0
West Yunan & East Tibet	-0.699	0.171	0
Fertile Crescent	0.372	0.159	0.019
Sava West India	0.211	0.089	0.018
Ganges of East India	1.076	0.2	0
West Africa	0.164	0.055	0.003
West African Savannah	-0.945	0.17	0
Sudanic savannah	-0.815	0.064	0
Ethiopian plateau	-0.915	0.061	0
Northern Lowlands of South America	3.82	0.733	0
Central/Southern Andes	0.793	0.27	0.003
Southwest Amazon	2.171	0.168	0
Mesoamerica	2.18	0.093	0
East North America	2.126	0.927	0.022
Model parameters: adjusted R-sq = 0.67, F-statistic = 140, df = 15,992, AIC = 1692.36			

Data S1. (separate file)

List of cultural traits with codes as recorded in the D-PLACE database (Original Code), and (if necessary) re-categorization of code traits for the present analyses (New Code). The Notes tab provides comments to guide interpretation and justify re-categorization (if necessary). Where a code is re-categorized with NA, societies with that code are excluded from the models concerning the particular cultural trait. A total of 67 traits were selected for the analyses, out of which we summarized correlates of cultural similarity for 54 traits (traits with non-viable models were excluded from the results).

Data S2. (separate file)

Variables reduced by PCA – input data associated with models analysing predictors for cultural sharing between pairs of societies. (a) The strength of indirect cultural transmission (potential for cultural similarity due to transmission between other close-by societies) is quantified by using the five nearest neighbours with available cultural data in the D-PLACE database. (b) The strength of indirect cultural transmission is quantified by using the ten nearest neighbours; if cultural data did not exist for at least three of these neighbours, the pair was discarded.

Data S3. (separate file)

Summary of sensitivity analyses for the ecological test of Diamond's hypothesis. For each trait, we ran the models using a tree from the posterior distribution (rather than a maximum clade credibility tree, like in the main analyses). Using samples from the posterior distribution rather than a consensus tree allows us to check the robustness of our results in terms of uncertainty of phylogenetic relationships between societies. We use 50 randomly selected trees from the posterior, and run all the steps for each trait. In DataS3 we report, for each trait and each predictor, the percentage of runs in which the effect of the predictor follows the expectations (negative for geo-environmental barriers to cultural spread and phylogenetic distance, positive for potential of similarity due to cultural transmission from other nearby societies), as well as the percentage of runs in which the predictor follows the expectations and it is significant ("_sign" columns). For example, for trait EA008, temperature barriers show a negative effect across all 50 runs, (TempPeffect = 1), and this effect is never significant (TemptPeffect_sign=0). All details of the sensitivity runs and building of DataS3 can be found on GIThub: https://github.com/angela-mc/AxisOrientationHypothesis/blob/main/scripts/EcologicalTest_edge/1getdata_EdgeTree100.R
